# Engineering antibacterial bioceramics: Design principles and mechanisms of action

**DOI:** 10.1016/j.mtbio.2024.101069

**Published:** 2024-04-27

**Authors:** Ngoc Huu Nguyen, Zufu Lu, Aaron Elbourne, Krasimir Vasilev, Iman Roohani, Hala Zreiqat, Vi Khanh Truong

**Affiliations:** a School of Biomedical Engineering, The University of Sydney, Camperdown, NSW, 2006, Australia; b Biomedical Nanoengineering Laboratory, College of Medicine and Public Health, Flinders University, Bedford Park SA 5042, Australia; c School of Science, RMIT University, Melbourne, Victoria, 3001, Australia

**Keywords:** Bioceramics, Implant-associated infection, Antimicrobial, Orthopaedic implant

## Abstract

The urgency to address skeletal abnormalities and diseases through innovative approaches has led to a significant interdisciplinary convergence of engineering, 3D printing, and design in developing individualised bioceramic bioscaffolds. This review explores into the recent advancements and future trajectory of non-antibiotic antibacterial bioceramics in bone tissue engineering, an importance given the escalating challenges of orthopaedic infections, antibiotic resistance, and emergent pathogens. Initially, the review provides an in-depth exploration of the complex interactions among bacteria, immune cells, and bioceramics in clinical contexts, highlighting the multifaceted nature of infection dynamics, including protein adsorption, immunological responses, bacterial adherence, and endotoxin release. Then, focus on the next-generation bioceramics designed to offer multifunctionality, especially in delivering antibacterial properties independent of traditional antibiotics. A key highlight of this study is the exploration of smart antibacterial bioceramics, marking a revolutionary stride in medical implant technology. The review also aims to guide the ongoing development and clinical adoption of bioceramic materials, focusing on their dual capabilities in promoting bone regeneration and exhibiting antibacterial properties. These next-generation bioceramics represent a paradigm shift in medical implant technology, offering multifunctional benefits that transcend traditional approaches.

## Graphical abstract

**Figure:**
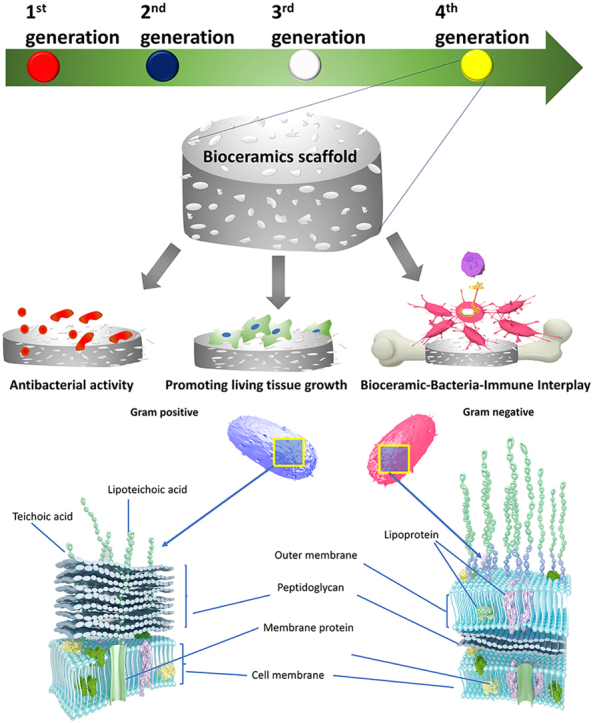

## 1 Introduction

The reconstruction of extensive bone defects caused by trauma, infection, osteoporosis, or resection of malignant tissue remains a major challenge in clinical treatment. Allografts and autografts suffer serious drawbacks, such as limited availability of healthy tissue (especially in older patients), morbidity at donor sites, and risks of infection and disease transmission. Metal and alloy implants possess the necessary mechanical strength, but are excessively rigid, not resorbable, and perform poorly in biocompatibility and bioactivity, often leading to aseptic loosening, infection, and other post-surgery complications. An excellent alternative that has emerged in recent years is bioceramic implants, known for their improved biocompatibility and significant bioactivity [[1], [2], [3]]. Despite their advantages, bioceramics in the clinic are still prone to bacterial infection, which significantly hampers their ability to prevent and combat implant-associated infections [[4], [5], [6], [7], [8]]. To fight against infection problems, bioceramics scaffolds with various antibacterial strategies are developed for bone repair and regeneration [9,10].

Implant-related infections are some of the primary factors related to premature implant failure. Strategies to treat implant infections primarily rely on administering antibiotics and surgical intervention. More than 200,000 bone transplants are performed annually worldwide to repair bone defects [11], with the global implant market predicting more than five percent annual growth and are expected to reach $145.6 billion in 2030 [12]. Implant-associated infections have been growing steadily, with a marked increase in the number of cases reported over recent years. According to the World Health Organization (WHO), antibiotic resistance is one of the top ten global public health concerns confronting humanity, and the fatalities related to this issue might grow from 700,000 to 10 million by 2050, surpassing cancer as the primary cause of death worldwide [[13], [14], [15]]. Biofilms are responsible for approximately 80 % of these infections, presenting a considerable challenge to healthcare systems and patient outcomes [16]. The development of bacterial antibiotic resistance and biofilm formation significantly diminishes the effectiveness of antibiotics [17,18]. Antimicrobials must penetrate the biofilm to successfully suppress bacterial growth in frequent infections. This escalating problem underscores the urgent need for innovative strategies to develop the next generation of bioceramics with suitable chemical and topological features. These are considered the most crucial prerequisites for biofilm formation to prevent and combat implant-associated infections. The most critical considerations for designing and manufacturing bioceramics are their resistance to microbial colonisation and biofilm formation. Developing bioceramics with integrated antibacterial properties is crucial in addressing the escalating challenges of implant-associated infections and antibiotic resistance, ultimately ensuring long-term success.

**Fig. 1:**
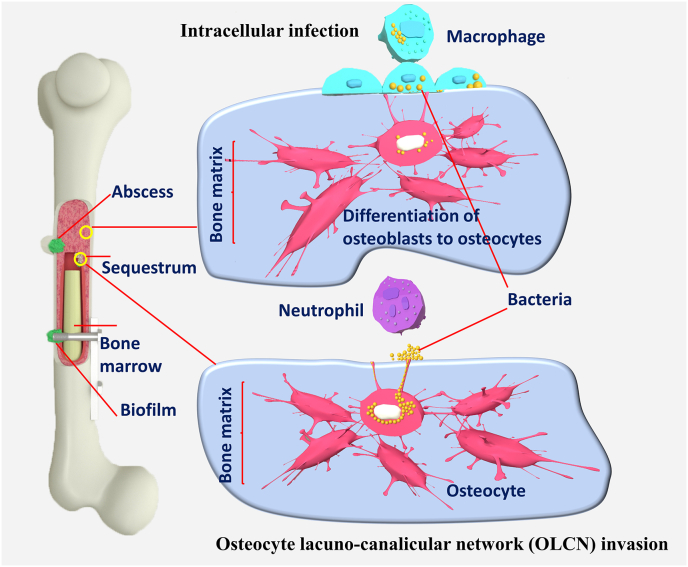
Bacterial strategies in osteomyelitis. Bacteria have a variety of pathogenic pathways. Bacteria persistence is most likely caused by intracellular infection of osteoblasts, osteoclasts, and osteocytes, and macrophages promote bacterial dispersion and multiorgan failure. Bacteria can evade host immune cells by invading the osteocyte-lacuna canalicular network, most commonly found within a sequestrum. Through diffusion constraints and metabolic variety, Bacteria biofilms on implant surfaces and necrotic bone confer immune cell and antibiotic resistance. Bacteria can be found in both long bones and soft tissue. In the center of an abscess, bacteria cells are detected, surrounded by a fibrous pseudocapsule and dead and live immune cells.

The top five bacteria causing implant infection, critical in the context of osteomyelitis, include *Staphylococcus aureus, Staphylococcus epidermidis, Pseudomonas aeruginosa, Enterobacter cloacae, and Escherichia coli* [19]. *S. aureus*, in particular, is notable for invading, colonising, and thriving within the bone, making it a particularly formidable pathogen in the case of osteomyelitis (Fig. 1). Gram-positive and Gram-negative bacteria have distinct cell shapes, biochemistry, and biomolecular mechanisms that enable them to survive and grow on the implant surface [20]. Furthermore, they are difficult to remove and resist the immune system and frequently cause opportunistic infections [21].

This review provides a comprehensive analysis of bioceramic-associated infections and the importance of developing the next generation of antibacterial bioceramics implants for orthopaedic reconstructive surgeries. An important focus will be the investigation of the complex interplay between various factors influencing bioceramic infection, such as protein adsorption, immune cell response, bacterial adhesion, and endotoxin release. Knowledge gained will inform the strategies to develop the next generation of antimicrobial bioceramics to prevent implant infection. By providing a comprehensive understanding of antibacterial mechanisms and strategies, we hope to pave the way for developing innovative solutions for preventing implant-associated infections and advancing next-generation bioceramics.

## 2 Advancements and challenges of bioceramics for bone tissue engineering

Key points:

• The importance of bioceramics in bone tissue engineering.
• The challenge of bioceramics in bone tissue engineering.
• Addressing challenges encountered in bioceramic infection, through design of antimicrobial materials, agent incorporation, composite creation, and advanced fabrication techniques.

The biocompatible, osteoconductive, and biodegradable properties of bioceramics and their composites make them ideal biomaterials used in bone tissue engineering [1,2]. Table 1 provides the comprehensive properties of bioceramics contributed in bone tissue engineering.

**Table 1:** Summary of the key aspects and significance of bioceramics.

Aspect	Significance	Ref
Nature of the bioactive bond	Apatite formation on the surface of bioceramics is pivotal in the direct bonding. The hydroxycarbonate apatite crystals were bound to layers of collagen fibrils generated by osteoblasts at the interface. The chemical bonding of the hydroxycarbonate apatite layer to collagen formed a strong interface connection. Five surface reactions at the surface occur: cation exchange and Si–OH group formation, on which amorphous calcium phosphate phase deposits, crystallising to HCA, which binds to collagen.	[22,23]
Bioceramics are biocompatible	Bioceramics closely mimic the composition of natural bone, promoting excellent compatibility with host tissue. They do not harm the natural tissues of the body and can be used for long-term implantation. Bioceramics provide a scaffold for bone cell attachment, migration, and proliferation, promoting new bone formation. Some bioceramics can induce osteogenic differentiation of stem cells, further enhancing bone regeneration.	[[24], [25], [26]]
Bioceramics can be designed to be resorbable	Bioceramics can be designed with controlled degradation rates, enabling gradual replacement with native bone tissue as new bone forms.	[1,[27], [28], [29]]
Bioceramics can be combined with other biomaterials	The incorporation of polymers (as composite) and metals(as coating), to create composite materials with enhanced properties for bone tissue engineering.	[25]
Bioceramics can be designed with different compositions and properties	They are versatile and customisable for various bone tissue engineering applications	[1,30]
Bioceramics can be fabricated into various shapes and sizes	Bioceramics are allowing for the creation of patient-specific implants that fit better and have improved stability using additive manufacturing techniques	[31,32,32]
Antibacterial properties	Some bioceramics exhibit antimicrobial properties, reducing the risk of infection in bone tissue engineering.	[33,34]
Cost-effectiveness	Bioceramics can be produced at a relatively low cost, making them a more accessible option for bone tissue engineering.	[35,36]

Bioceramics can be made from different types of materials, including bioactive glasses (BG), calcium phosphates, and silicate-based ceramics, each with various advantages depending on the application [56,57]. This adaptability enables the development of bioceramics with customised characteristics to satisfy the needs of various clinical circumstances. Advances in material science and fabrication technologies, such as 3D printing, have enabled the creation of bioceramic-based scaffolds with controlled porosity, mechanical strength, and degradation rates, allowing for better integration with the host tissue and improved tissue regeneration [58,59].

Infection-related issues associated with bioceramics represent an important drawback to their successful clinical application. One of the primary concerns is bacterial adhesion and colonisation on the surface of bioceramic implants, which can contribute to biofilm formation and difficult to treat persistent infections. In addition, the widespread usage of antibiotics raises concerns regarding the emergence of antibiotic-resistant bacteria [60], creating difficulty for long-term infection control strategies. Infections can also hinder the osseointegration of bioceramic implants and induce an adverse immune response, resulting in implant failure [61]. Furthermore, balancing the antimicrobial properties, mechanical properties, biocompatibility, and bioactivity of bioceramics can be difficult because modifying one property can deleteriously affect the others. It is essential to overcome these obstacles to develop infection-resistant bioceramic implants with the desired biological and mechanical properties.

**Fig. 2:**
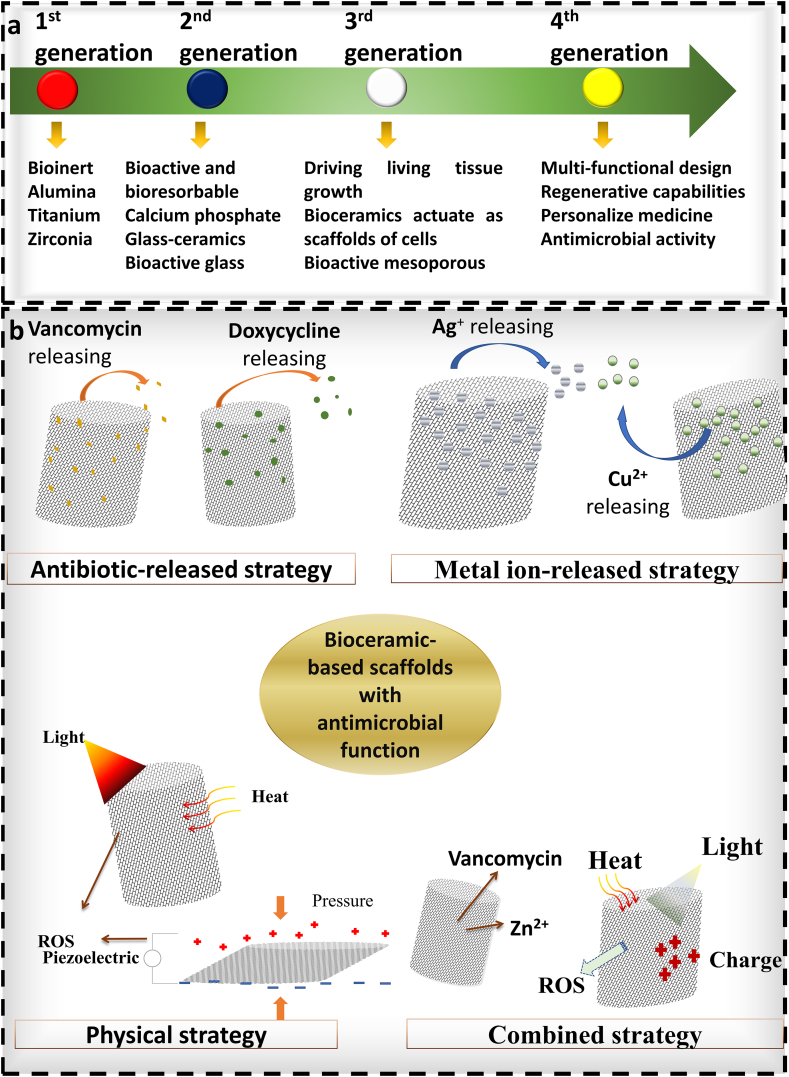
Evolution and advancements of bioceramics. (a) A schematic overview of the fourth generations of bioceramics, illustrating their progressive development. (b) The innovations and achievements in bioceramics-based scaffolds with diverse antibacterial properties for addressing bone implant-associated infections and bone deformities. These include scaffolds employing drug-induced, ion-mediated, physically activated, and combined antibacterial strategies to enhance their effectiveness in promoting bone healing and preventing infections.

Designing bioceramic materials with inherent antimicrobial properties, such as incorporating antimicrobial ions (e.g., silver (Ag), copper (Cu), zinc (Zn), etc.) or developing ceramics with specific surface topographies that discourage bacterial adhesion, is one approach, as shown in Table 2. In the other approach, antimicrobial peptides or agents can be incorporated into the material so that they can be released in a controlled and sustained manner, providing local antibacterial activity without causing systemic adverse effects. Additionally, combining bioceramics with other biomaterials to create composites may enhance the equilibrium between antimicrobial properties and other desirable characteristics, such as mechanical strength and biocompatibility [62,63]. Complex structures with customised surface properties that inhibit bacterial adhesion and biofilm formation can also be fabricated using advanced fabrication techniques, such as 3D printing. Exploring these innovative methods allows researchers to tackle the issues associated with infection-related complications in bioceramic applications. Fig. 2 illustrates the progression of bioceramics, highlighting the innovations and accomplishments in bioceramics-based scaffolds that possess varied antibacterial properties. These advancements are specifically designed to combat bone implant-associated infections and correct bone deformities. Various strategies, such as drug-induced, ion-mediated, physically activated, and combined antibacterial methods, are employed in these advanced scaffolds to enhance their effectiveness in promoting bone healing and preventing infections.

**Table 2:** Metals doped bioceramics for antibacterial activity.

**Metal**	**Bioceramics**	**Antibacterial activity**	**Mechanisms**	**Application**	**Ref**
Silver	HA/chitosan nanocomposite coatings, coating	Broad-spectrum against bacteria, fungi, and viruses	Release of Ag^+^ ions that damage bacterial cell membranes and intracellular biomolecules induce oxidative stress and ROS production.	Orthopaedic and dental materials, bone-related implants, wound dressings	[37,38]
Copper	Calcium phosphate and tricalcium phosphate	Effective against *E. coli, S. aureus*	Membrane permeability alteration, protein function disruption	Bone graft substitutes, dental implant coatings, bone regeneration implants,	[39,40]
Zinc	Bioactive Glass, Zinc oxide nanocrystals, Zn-modified HA	Gram-positive and Gram-negative bacteria	Inhibition of microbial adhesion interferes with bacterial cell membrane and intracellular processes and induces oxidative stress and ROS production. Bone repair, multifunctional bone implant	Bone repair, multifunctional bone implants, Coating for metal implants	[41,42]
Magnesium	MgO nano-layer, magnesium-substituted HA	Antibacterial and biofilm prevention	Alkaline environment creation, competitive inhibition of calcium binding	Orthopaedic applications, implant coatings	[43,44]
Gallium	HA	*P. aeruginosa*, MRSA	Iron metabolism interference, membrane damage	Osteoconductive scaffolds, infection-resistant surfaces	[45,46]
Cerium	Zirconium Oxide	Wide range of bacteria including multi drug resistant strains	Oxidative stress induction, membrane disruption	Prosthetic devices, antimicrobial films	[47,48]
Cobalt	HA	Various bacterial strains	Co ion release leading to antibacterial activity, angiogenesis promotion	Vascular stents, bone tissue scaffolds	[49]
Bismuth	Calcium Phosphate	Effective against a broad range of pathogens, including *H. pylori* and *E. coli*	Bi ions disrupt enzyme activities and bind to bacterial proteins, inhibiting their functions	Used in gastrointestinal devices, dental fillings, and as a radiopaque material	[50,51]
Strontium	Calcium Phosphate	*S. aureus*,	Osteoinduction, ionic substitution affecting bacteria metabolism	Dental applications, bone defect fillers	[52,53]
Manganese	Bioactive glass, nano HA	*E. coli, Shigella dysenteriae, S. aureus*	Increase pH in medium, ROS production	Bone implant	[54,55]
Irion	Nano HA	*Shigella dysenteriae*	ROS production		[55]

The manufacturing techniques of ceramics, such as 3D printing, play a crucial role in determining their antimicrobial properties. Firstly, the 3D printing process affects the surface characteristics of the final products, which in turn influences their antimicrobial efficacy [64]. Secondly, both the microstructure and surface characteristics, shaped by the production method, are critical factors in the ability of bioceramics to resist microbial colonisation [65].

**Fig. 3:**
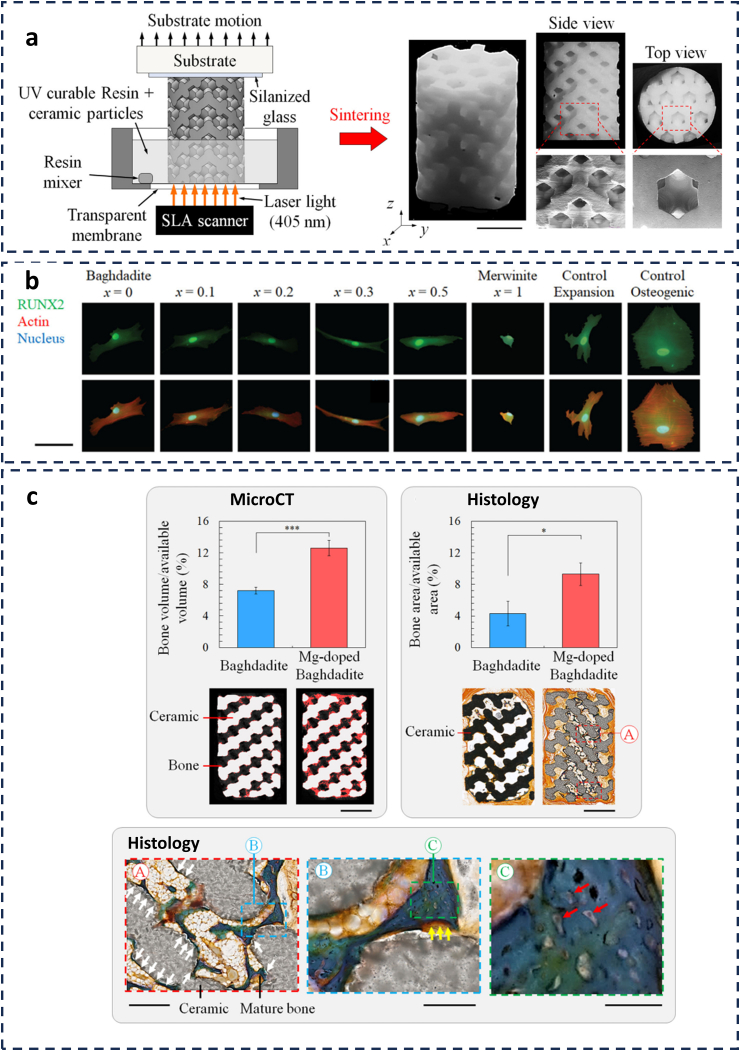
Advancements in bioceramic scaffold magnesium doped baghdadite fabrication and cellular response evaluation. (a) A multi-panel figure showcasing the SLA-based fabrication process of bioceramic scaffolds. (b) The expression of osteogenic marker RUNX2 in cells cultured on different scaffold compositions. (c) The comparative analysis of bone volume and histological integration between standard and magnesium-doped baghdadite scaffolds. (Adapted with permission from Ref. [32]).

Advancing next-generation bioceramic scaffolds requires a multidisciplinary approach that integrate various cutting-edge techniques and methodologies (Fig. 2, Fig. 3). This approach encompasses the use of computer-assisted graded pore design to optimise scaffold architecture, the advanced synthesis of ceramic powders for tailored material properties, and the employment of 3D printing technologies for precise and customized fabrication [66]. In addition, the incorporation of antibacterial agents and surface modifications can improve the resistance of the scaffold to infection while promoting cell formation and tissue integration [67,68]. *In vivo* studies are essential for evaluating the performance of these novel bioceramic scaffolds, ensuring that they meet the desired biocompatibility (Fig. 3). Calcium silicate-based bioceramics including mineral trioxide aggregate (MTA), have been evaluated in dental applications through subcutaneous implantation models, demonstrating encouraging outcomes against oral pathogens [69]. Similarly, HA bioceramics doped with antimicrobial ions such as silver (Ag^+^) and zinc (Zn^2+^) were evaluated in rat tibia osteomyelitis models and rabbit femoral defect experiments to assess their effectiveness in preventing infections and enhancing bone regeneration [70]. By integrating these strategies, researchers can successfully design and develop bioceramic scaffolds for orthopaedic and dental implant applications, effectively addressing the ongoing challenges in these fields.

## 3 The complex interactions in bioceramic-associated infections: bacteria, bioceramics, and immune cells

Key points:

• Bacteria that can adapt to almost any bioceramics surface and thrive in a hostile host environment rely on their ability to adhere quickly and to survive.
• Biofilm development on implant surfaces protects germs and promotes infection persistence.
• The immune system of host reacts to both germs and the bioceramic implant surface

**Fig. 4:**
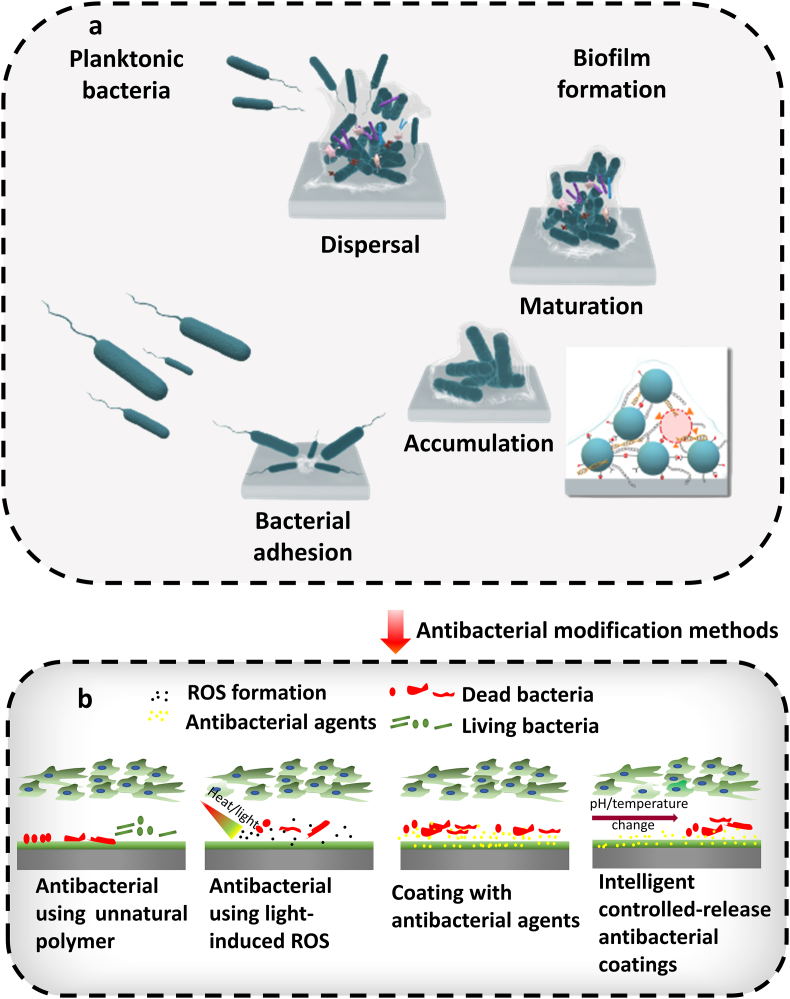
The schematic illustration of stages of biofilm formation and main antibacterial modification methods. (a) A model of biofilm formation with common characteristics, including bacterial adhesion, accumulation, maturation, and dispersal. Planktonic cells interact and adhere to the surface of the bioceramics. Bacteria cluster together and form microcolonies due to intercellular contacts mediated by adhesins and cell wall proteins. Fibronectin-binding proteins (FnBPs) build a bridge between fibronectin (Fn) molecules, promoting bacterium aggregation. Extracellular polymeric compounds are produced as part of the biofilm maturation process, during which the biofilm matrix gradually thickens, and larger bacterium aggregations called towers form. Biofilm production is facilitated by the expression of the polysaccharide intercellular adhesin and the release of extracellular DNA obtained from bacteria and dead host cells. (b) Schematic representation of various antibacterial modification strategies for bioceramics. These strategies include incorporating antibacterial activity through the use of unnatural polymers, which can be designed and engineered to target and disrupt bacterial cell walls or membranes; utilising light-induced reactive oxygen species (ROS) generation to damage bacterial cells and inhibit their growth; coating bioceramic surfaces with antibacterial agents, such as metal ions, to enhance their antimicrobial properties; and implementing intelligent controlled-release antibacterial coatings that can respond to specific stimuli, allowing for a targeted and sustained release of antimicrobial agents. These approaches aim to improve the antibacterial performance of bioceramics in various medical applications.

Bacterial attachment to the surface of biomaterials is the initial step prior to biofilm formation [71]. When bacteria colonise a surface, they create colonies, eventually expanding into huge heterogeneous structures called biofilms. The stages of biofilm growth are bacterial adhesion, microcolony establishment, biofilm maturation, and biofilm dissipation (Fig. 4). Biofilms protect microorganisms from environmental changes, antibiotic dosages, host immunity, and assist horizontal gene transfer [72].

The bacteria initiate intercellular communication within the biofilm and rapidly regulate gene expression, allowing for temporal adaptations like phenotypic diversity and low-nutrient survival [73]. Bacterial biofilms are difficult to remove, causing repeated infections that affect bioceramics performance, healing, and disease progression [74]. At the molecular level, biofilm formation begins with the binding of adhesins and cell wall proteins like FnBPs to substrates, resulting in the congregation of bacteria and the subsequent production of extracellular polymeric compounds [75]*. Staphylococci* possess a variety of surface-associated adhesins involved in both initial biofilm cell attachment and intercellular adhesion throughout biofilm maturation [76]. Covalently bound cell wall proteins, non-covalently binding proteins, and non-protein components contribute to *Staphylococcal* adhesion and biofilm development [77]. *S. aureus* produces two FnBPs, FnBPA and FnBPB, encoded by the genes *fnbA* and *fnbB* [78]. The FnBPs feature an N-terminal region, and these domains are involved in the binding of fibrinogen and elastin. The binding of FnBPs to Fn promotes bacterial invasion into epithelial, endothelial, and keratinocyte cells [79]. For instance, the *Staphylococcus aureus* genome encodes more than twenty adhesins [80]. The pathophysiology of *S. aureus* infections is characterised by biofilm formation, which binds and anchors to a substrate. Then, adhesins and cell wall proteins like FnBPs that bind Fn molecules cause *S. aureus* to congregate and form a biofilm. The maturation stage involves increased extracellular polymeric compounds produced by the biofilm matrix and increased *S. aureus* aggregation. For final dispersal, *S. aureus* cells return to the planktonic stage [81]. A previous study reported that a double knockout of *fnbA* and *fnbB* in *S. aureus* resulted in a loss of fibronectin binding and the ability to produce biofilms on microtiter plates and shear flow conditions. The complementation of fnbA or fnbB alone on a plasmid restored these characteristics and the capacity to agglutinate *S. aureus* [82].

Biofilms can be single or mixed species and exhibit distinct multicellular behaviours, making them difficult to eradicate and contributing to the problem of antibiotic resistance. These bacteria reproduce, mature, and thrive on the surface of bioceramics, where resources are plentiful, resulting in increased antibiotic resistance in large microbial populations [83]. For instance, fluconazole and vancomycin sensitivity decreased in a polymicrobial biofilm, including *Candida albicans* and *Staphylococcus epidermidis*, respectively [84]. The extracellular polymeric matrix provides the biofilm with increased antibacterial resistance. Phagocytosis, opsonisation, physical stress, and antibacterial diffusion are all inhibited by this gel-like matrix [85]. As a result, microbes deep within the surface of gadgets continue to thrive. An ionically charged extracellular matrix can interact electrostatically and inhibit the action of a wide variety of cationic antimicrobials, such as aminoglycosides [86]. Additionally, biofilms provide an isolated habitat for infections to share genetic information via plasmids. The spread of these plasmids between species exacerbates the problem of antibiotic resistance [87].

Therefore, to avoid the biofilm formation, it is critical to follow all aseptic surgery standards carefully. Another method of reducing the chances of bacterial adherence is to develop novel materials or improve the surface of implanted medical devices to prevent them from attracting hazardous biofilm infections [87]. Multifunctional coatings on a zirconia surface, a nanostructured surface, and controlled antibiotic release can significantly contribute to this objective [88].

### 3.1 Host immune response to bioceramic-associated infections and bone regenerations

The host immune system reacts to both germs and the surface of an implant, identifying it as a foreign body [89]. This reaction activates the coagulation cascade, complement system, platelets, and immune cells, mainly neutrophils [90]. The constant release of reactive oxygen species from bioceramics causes metabolic stress and depletion of oxidative resources, reducing ability of neutrophils to fight germs [91]. Several studies indicate innate immune cells as important anti-infective biomaterial targets. In addition, a significant reduction in bacterial numbers, suggesting a target of 2-3 log reduction to demonstrate efficacy, the immune system can clear any remaining bacteria [92]. Innate immune cells adhere to biomaterials within hours, while lymphocytes do not [93]. Neutrophils and macrophages are innate immune effectors against planktonic *Staphylococci* species [94]. Their ability to directly kill microbes is necessary for infection clearance to be successful. The failure of the host to eliminate the bacterial burden is commonly related to mortality. Macrophages and neutrophils control the balance of inflammation and tissue repair required for biomaterial integration [95].

#### 3.1.1 Innate immunity in bioceramic infection defense and bone regeneration

Neutrophils are activated immediately and are the first cell type to gather around a biomaterial in the innate immune system. These cells are responsible for eliminating cellular debris and pathogens by phagocytosis, producing reactive oxygen species, degranulation, and generating pathogen-encapsulating neutrophil extracellular traps (NETs) [96]. The inflammatory response is maintained by neutrophils releasing cytokines (IL-1) and chemokines (MCP-1 and CXCL1) that attract monocytes. Neutrophils are vital in the fight against *Staphylococci* infection around the implant [97]. Many potent antimicrobial proteins and components are found in neutrophil intracellular granules, making them highly effective in killing bacteria intracellularly. The fact that decreased neutrophil activity around the implant increases the risk of biomaterial infection highlights the importance of maintaining normal neutrophil function near the biomaterial [98]. Furthermore, the activation of neutrophils is enhanced by biomaterial-specific processes. First, extracellular matrix/blood proteins and complement factors coat the biomaterial, providing additional sites for neutrophil adherence and activation [99]. Second, accumulating evidence shows that surface properties of biomaterials affect neutrophil activity [100]. For example, in this process, the adsorption of blood/extracellular matrix proteins and complement components activates neutrophils. They produce ROS, degradation enzymes, and NETosis in response to local trauma or pathogen-induced proinflammatory stimuli. This is due to metabolic fatigue, HDP deactivation, increased ROS generation and NETosis, and/or inflammasome activation [98] linked to decreased bacterial uptake and death [101]. Neutrophils, on the other hand, produce NETs to extracellularly trap and kill germs, which is the final stage of an active neutrophil death process called NETosis [102]. It is impacted in the presence of a biomaterial and is thought to be a major contributor to the destructive inflammation associated with non-immunocompatible biomaterials, resulting in impaired neutrophil phagocytosis and tissue healing. Therefore, anti-infective strategies should attempt to reduce neutrophil-mediated inflammation caused by unregulated ROS and NET generation while restoring or increasing their anti-infective effects [98].

Macrophages, as professional phagocytes, provide a second line of defence against any bacterial problems in the region of the biomaterial [98,103]. Macrophages release various cytokines and growth factors that tightly regulate the osteogenic function of mesenchymal stem cells [104]. Monocytes develop from myeloid progenitor cells, which differentiate into monoblasts, pro-monocytes, and ultimately monocytes. The presence of colony-stimulating factors released by stromal cells in the blood and tissues induces this cell development [105]. After biomaterial implantation, monocyte-derived inflammation macrophages are recruited and undergo phenotypic alterations to adapt to the local microenvironment. The major macrophage subtypes are classified as M1 or M2 macrophage subtypes [106]. The M1 macrophage activated by strong inflammatory stimuli like toll-like receptor ligands or interferon-γ (IFN-γ) is responsible for proinflammatory cytokine production, phagocytosis, and antigen presentation [107]. M2 macrophages develop in response to IL-4, IL-13, or IL-10 stimulation and are principally important for moderating the inflammatory response and coordinating tissue regeneration [108]. However, the proinflammatory M1 signature of macrophages in response to bacterial infection [109]. Numerous effectors contribute to M1 enhanced microbicidal activity of macrophage and mostly involve the uptake of bacteria within the degradative phagolysosome, a process that requires the formation of ROS and nitric oxide NO. Additionally, several genes involved in M1 polarisation are increased in response to bacterial infections, including those encoding the cytokines tumour necrosis factor (TNF)-, IL-6, IL-12, and IL-1, as well as chemokines CCL2, CCL5, and CXCL8 [109]. Finally, an M1 macrophage characteristic is its enhanced ability to educate adaptive immunity via antigen presentation [110]. Notably, bioceramics have been shown to be critical regulators of macrophage immunomodulation via ion products such as SiO_4_^4−^, Ca^2+^, and Mg^2+^ [111]. Macrophages activated with bioceramics secreted much more cytokines, chemokines, and proteases that govern inflammation and subsequent osteogenesis/angiogenesis. The paracrine route mediated by bioceramic-induced macrophage exosomes has not been fully understood in bioceramic-mediated bone repair.

Dendritic cells are significant because they play an essential role in initiating and regulating immune responses [112]. Dendritic cells are the primary antigen-presenting cells, linking the innate and adaptive immune systems. They capture process, and present antigens to T cells, triggering specific immune responses against pathogens. For instance, bioactive glass has been observed to influence the maturation and activation of dendritic cells positively [112,113]. Strontium (Sr)-containing BG can promote dendritic cell maturation, leading to an increase in the expression of co-stimulatory molecules and pro-inflammatory mediators [114]. This enhanced response can improve the capacity of the host to manage infection and facilitate healing.

Natural killer (NK) cells play an important role in bioceramics infection and immune response due to their ability to recognise and prevent infected cells without prior sensitisation. They are part of the innate immune system and contribute to the initiation of the defence against infections. In the case of bioceramics-associated infections, NK cells can aid in limiting the dissemination of the infection and promoting a quicker resolution of the inflammatory response. Certain bioceramics, such as silicon-substituted calcium phosphate (Si–CaP), have been demonstrated to stimulate NK cell activity, thereby increasing their cytotoxic potential against infected cells. This increased NK cell activity may contribute to a more effective immune response against pathogens, preventing further infection and promoting tissue integration and healing around implanted bioceramic materials.

#### 3.1.2 Bioceramics and adaptive immunity: modulating T and B lymphocyte responses for improved infection defense and bone regeneration

T lymphocytes are a heterogeneous group of immune cells that include T helper cells, cytotoxic T cells, and regulatory T cells, each of which performs a distinct function in the immune response [115]. When implanted, bioceramics interact with the host immune system, including T lymphocytes [116]. T lymphocytes play an essential role in adaptive immunity, which is essential for protecting the body from pathogens and promoting tissue repair [117]. Understanding the interaction between bioceramics and T lymphocytes can shed light on how these bioceramics influence the immune response and contribute to the prevention and control of implant-associated infections.

Bioceramics can modulate T lymphocyte activation and proliferation, which influences the overall immune response against pathogens and plays a role in tissue regeneration. For instance, HA promotes CD4^+^ T cell proliferation and Th1 cytokine secretion, such as interferon-gamma, thereby aiding in the control of infection [116,118]. Moreover, bioceramics can affect the ratio of regulatory T cells (Tregs) to effector T cells, thereby preserving immune homoeostasis and preventing excessive inflammation [119,120]. Understanding the relationship between bioceramics and T lymphocytes can guide the development of bioceramics with enhanced antimicrobial and osteoinductive properties, thereby promoting a balanced and effective immune response against bioceramics-associated infections.

B lymphocytes are critical because they are vital in the adaptive immune response to infections [117]. Antigens on pathogens can be recognised and bound by B cells, resulting in the production and secretion of antibodies. These antibodies can neutralise pathogens, stimulate immune cell phagocytosis, and activate the complement system, all contributing to pathogen clearance. Bioceramics, such as BG, can influence B cell function by augmenting antibody production and humoral immunity [90,117]. This may result in enhanced pathogen clearance and contribute to the overall immune response of the host against bioceramics-associated infections. By producing specific antibodies that recognise and neutralise pathogens, B cells play a crucial role in the adaptive immune response. Some bioceramics can enhance the production of pathogen-specific antibodies by stimulating B cell activation and differentiation into antibody-secreting plasma cells [121]. This increased antibody production can aid in opsonising and neutralising bacteria, thereby facilitating their removal by phagocytic cells and preventing colonisation on bioceramic surfaces. In addition, the humoral immune response can contribute to the formation of immunological memory, which can offer long-term protection against recurrent infections.

#### 3.1.3 Bioceramic-mediated modulation of host immune response for enhanced bone regeneration

Bioceramics can modulate the expression of numerous inflammatory cytokines and chemokines, influencing the immune response to infection and tissue repair of the host. For example, BG has been shown to stimulate the production of pro-inflammatory cytokines, including interleukin-1 beta (IL-1) and tumour necrosis factor-alpha (TNF-α) [122]. These cytokines enhance the capacity of the host to control infection by promoting inflammation and recruiting immune cells to the infection site.

On the other hand, HA has been shown to inhibit the release of pro-inflammatory cytokines while fostering the production of anti-inflammatory cytokines such as interleukin-10 (IL-10) [123]. This modulation aids in resolving inflammation and promoting tissue healing by fostering an environment more conducive to tissue repair and regeneration. The balanced expression of inflammatory and anti-inflammatory cytokines assures an efficient immune response to bioceramics-associated infections while minimising the risk of tissue damage caused by excessive inflammation.

Bone infection disrupts the balance between bone-forming osteoblasts and bone-resorbing osteoclasts, resulting in pathological bone loss and impaired healing [124]. Under their osteoimmunomodulatory properties, bioceramics can assist in restoring this equilibrium and fostering bone regeneration. The regulation of cytokines and chemokines is a mechanism by which bioceramics modulate the osteoimmune environment. HA and CaP can stimulate the production of anti-inflammatory cytokines, such as interleukin (IL)-10 and transforming growth factor-beta (TGF-β) [125,126], while inhibiting the production of pro-inflammatory cytokines, such as tumour necrosis factor-alpha (TNF-α) and interleukin-6 (IL-6) [127,128]. This modification to the cytokine profile can reduce inflammation and promote bone healing.

Moreover, bioceramics can affect immune cell differentiation and function. CaP and HA or HA, for instance, have been shown to promote the differentiation of anti-inflammatory M2 macrophages while inhibiting the differentiation of pro-inflammatory M1 macrophages [129,130]. This modification of macrophage polarisation can contribute to an osteoimmune environment that is more conducive to bone regeneration. Such effects underscore the potential of bioceramics to interact beneficially with the host immune system, enhancing both the biological integration and functional outcomes of bone implants.

## 4 Antibacterial bioceramics strategies in bone tissue engineering

Key points:

• Bioceramics doped with metal ions can prevent bacterial infections in medical implants.
• Understanding the mechanisms by which metal ions disrupt bacterial growth is crucial for developing effective antibacterial materials.
• Surface modification techniques, such as nanopatterning and coatings, can prevent bacterial adhesion and biofilm formation on implant materials.
• Smart antibacterial bioceramics with stimulus-responsive mechanisms show promise for preventing implant-related infections.

Antibacterial bioceramics are crucial in bone tissue engineering, ensuring successful implant integration and infection prevention. Various strategies have been developed to achieve this, including antibacterial adhesion, incorporation of metal ions, nanotechnology applications, and smart responses to bacterial presence. By employing these innovative approaches, researchers can create bioceramic materials with enhanced antimicrobial properties, promoting tissue regeneration and reducing the risk of infection. Ultimately, these advancements contribute to regenerative medicine and improve the clinical success of bioceramic-based implants.

### 4.1 The antibacterial activity of ions released from doped bioceramics

**Fig. 5:**
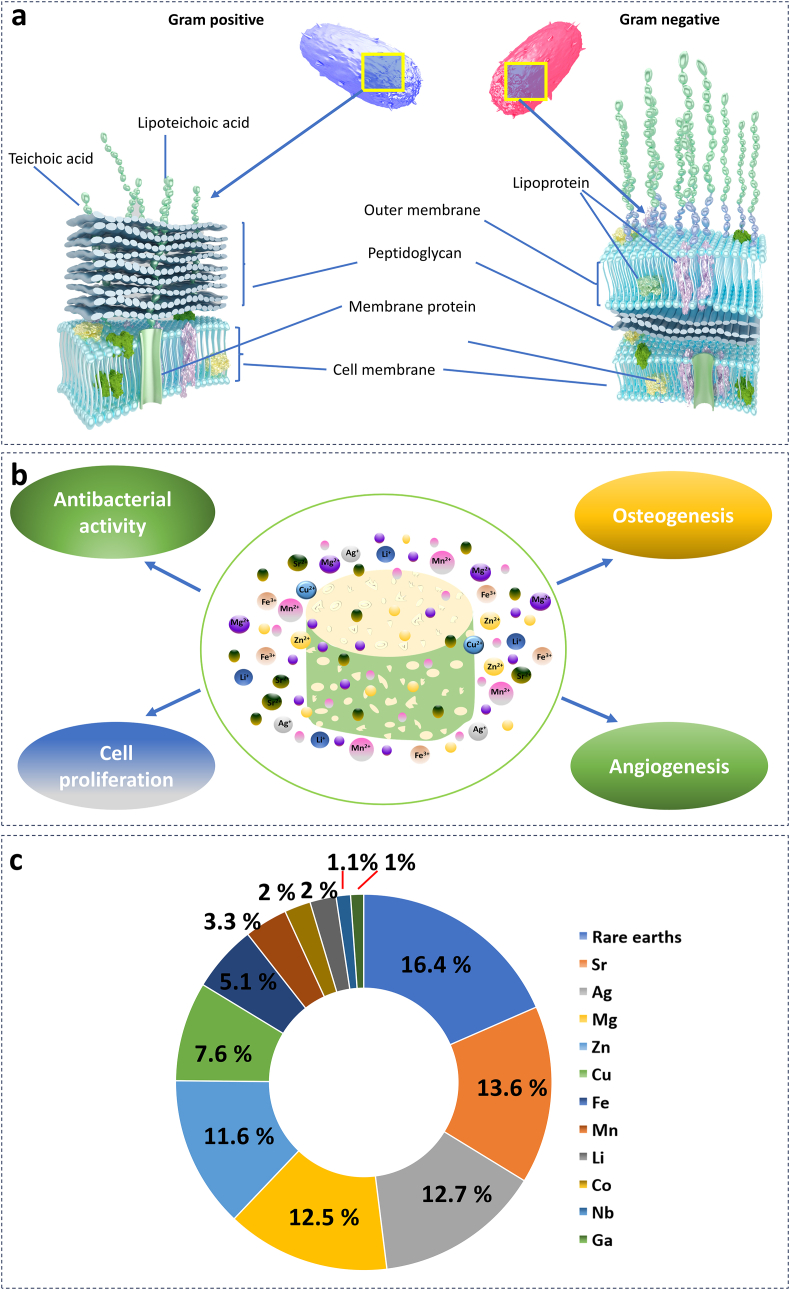
Recent developments and mechanisms of antimicrobial toxicity of metal and metal ions in the field of metal-doped bioceramics for bone tissue engineering. (a) Structure of Gram-negative and Gram-positive where different strategies target different components for antibacterial activity. (b) Biological responses to metal ions on bioceramics matrices. (c) Different metal ions doped in bioactive glasses and bioceramics (adapted from ref [136]).

In comparison to other strategies for enhancing the antibacterial properties of bioceramics, metal-doped bioceramics have gained popularity for several reasons. First, metals such as Ag, Cu and Zn display a broad spectrum of antimicrobial activities against a variety of bacterial strains, including Gram-positive and Gram-negative species [[131], [132], [133], [134]]. This broad-spectrum activity renders metal-doped bioceramics highly effective against a variety of infections. In addition, the combination of metal ions and bioceramics can produce synergistic antibacterial effects, thereby enhancing the antimicrobial performance of bioceramics [135]. This synergy produces an antibacterial action that is effective and long-lasting, preventing bacterial adhesion, colonisation, and biofilm formation on the surface of medical devices. For example, Zn and Ag-doped HA coatings exhibited synergistic antibacterial activity against methicillin-resistant *Staphylococcus aureus* (MRSA). Zn and Ag combined effect was greater than their individual effects when administered separately. A further benefit is the regulated release of metal ions. Metal ions may be incorporated into bioceramics in various ways, allowing for the release of ions over time. This controlled release ensures a sustained antibacterial effect, thereby minimising the risk of bacterial resistance development and potential side effects. Fig. 5 indicates that incorporating these elements into bioceramics may increase their biological activities.

In certain instances, incorporating metal ions into bioceramics can enhance their mechanical properties, such as hardness, fracture toughness, and wear resistance. In orthopaedic applications, where the mechanical performance of the implant is crucial to its long-term stability and success, this can be advantageous.

In addition, numerous metal ions, such as Ag^+^, Cu^2+^, and Zn^2+^ have demonstrated biocompatibility and pose minimal risk to human tissue. By doping bioceramics with these metal ions, the resultant material retains its biocompatibility, ensuring its suitability for medical applications. Therefore, metal-doped bioceramics offer many benefits over other antibacterial modification strategies. These advantages make them an appealing option for enhancing the antibacterial performance of bioceramic materials in a variety of medical applications.

#### 4.1.1 Antibacterial activity of copper and copper ions

Cu is a very important element that plays a role in a lot of different enzymatic systems and helps make cytochrome oxidase and superoxide dismutase, two enzymes that are important for cellular respiration (antioxidant defence) depending on their oxidation state [137]. Firstly, Cu^2+^ is attracted to cysteine and has a strong affinity for thiols, which is the only thiol-containing amino acid in the body. However, when Cu^2+^ is bound to cysteine, it goes through a process of homeostasis. When Cu^2+^ binds to cysteine, it is reduced to Cu^+^, which makes cystine the oxidised dimer of cysteine [138,139]. Second, Cu-catalysed reactions can oxidise biomolecules, such as reduced glutathione (GSH). GSH has a strong affinity for covalently linking Cu^2+^ and other bacterial proteins, such as cysteine-rich metallothioneins. These proteins have an unusually high number of cysteine residues in their sequence and most likely have a function in metal toxicity defence [140]. In addition, bacterial cells have copper efflux pumps, such as the CopA, a P-type copper efflux ATPase that keeps the intracellular content of copper low. Other Cu-binding proteins include the CueO multi-Cu oxidase and the CusCFBA multicomponent efflux transport system, contributing to intracellular Cu homeostasis and bacterial cell defence [141,142]. In summary, Cu is an important element, and bacteria can keep it in balance by avoiding its toxicity inside the body. Therefore, high concentrations of copper ions can damage important enzymes, like those that help bacteria get their energy from the main source of energy in the respiratory electron transport chains, which are made up of cysteines.

The precise mechanism of antimicrobial activity of copper ions remains unclear, but several lines of likely interconnected pathways have been proposed, and it is expected that a sequence of different pathways leads to bacterial cell death, including disruption of the cell membrane, intracellular alteration of biochemical processes, and induction of DNA damage. The first mechanism, the antibacterial properties of copper ions, have been shown to attach to bacterial cell walls, impairing the integrity and function of the cell membrane and related proteins. For example, copper and ion binding to phospholipids may modify the physicochemical properties of the membrane, reducing membrane fluidity and flexibility. Additionally, this may enhance oxidative stress at the membrane surface due to increased hydroxyl radicals and may disrupt the electron transfer chain by direct or indirect contact with the quinone pool [143]. A previous study showed that the release of copper ions from metallic surfaces results in severe membrane damage, as evidenced by the complete breakdown of the membrane into lipids in *E. coli* following treatment with a soluble copper salt. These findings show that the oxidation of membrane lipids is the fundamental mechanism by which copper ions kill bacteria, either by disrupting and degrading the membrane or impeding cell growth and division upon uptake [144].

#### 4.1.2 Antibacterial activity of silver and silver ions

Ag^+^ is well known to be toxic to bacteria, viruses, fungi, and various other organisms while posing little or no toxicity to humans [[145], [146], [147]]. The most common mechanism of action for Ag compounds is widespread disruption of cellular functions as a result of direct damage to the cell membrane or intracellular biomolecules and induction of oxidative stress as a result of metal-mediated ROS production, culminating in the formation of free radicals and widespread cellular damage [148][149].

The electrostatic attraction between the negatively charged bacterial surface and the positively charged Ag^+^ to adhere to the cell wall and membrane is the primary mode of action of this metal ion. The charge interaction between the bacterial cell and the Ag^+^ can affect the cell surface zeta potential, increasing membrane permeability, depolarisation, and decreasing respiratory potential [148]. Finally, a complete loss of membrane integrity causes irreversible cell damage and death. Ag^+^ rapidly reacts with the sulfhydryl groups on the bacterial cell membrane, exchanging the terminal hydrogen atom to form a stable S–Ag bond and thus completely inhibiting the respiratory chain, electron transfer, protein secretion, and lipid biosynthesis. A recent study established that Ag ^+^ primarily targets the bacterial membranes of *E. coli* and *P. aeruginosa* [150].

For example, the previous study showed Ag^+^ substituted HA bioceramics have superior osteoconductivity and high antibacterial activity and proposed a method for the antibacterial activity of such bioactive ceramics to be induced by the stern interface [151]. Due to the trace dopant Ag^+^ enrichment in the stern layer of the electric double layer at the negatively charged surface of Ag-HA bioceramics, the concentration of Ag^+^ at the stern interface of Ag-HA bioceramics is almost 5 times that in the bulk solution during this antibacterial process. HA with trace Ag^+^ generates a positive shift in the zeta potential and an increase in hydrophilicity, which may aid in inhibiting bacterial development.

#### 4.1.3 Antibacterial activity of strontium

**Fig. 6:**
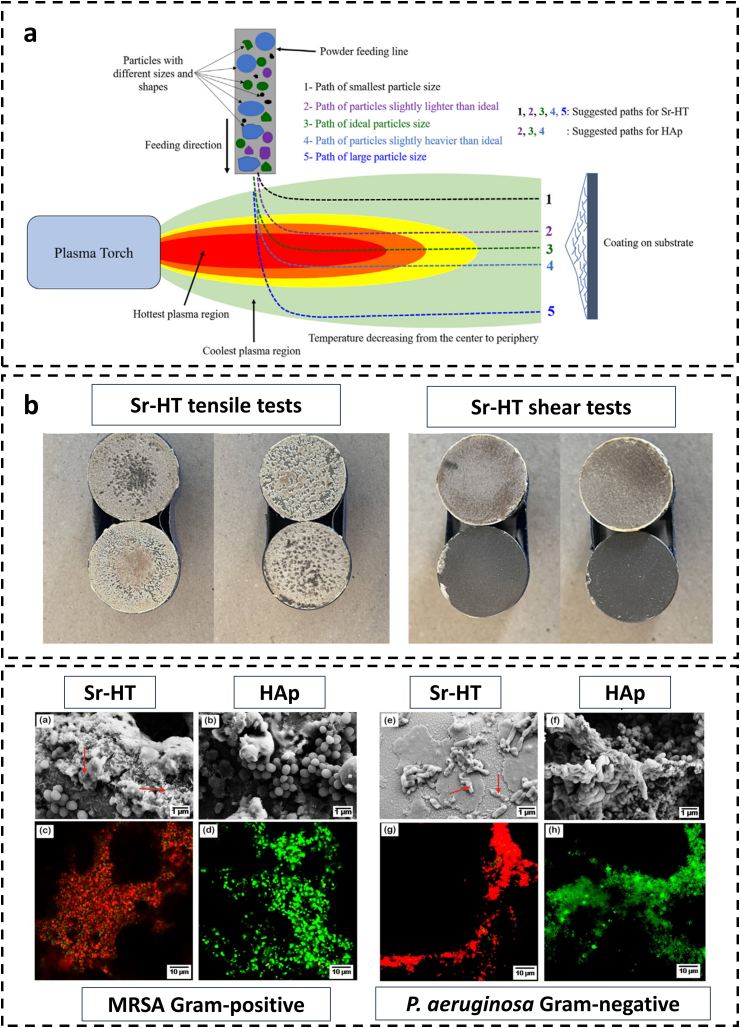
Antibacterial efficacy Sr-HT. (a) Illustrates the trajectory of bioceramic particles of various sizes and shapes within a plasma spray coating process, identifying optimal paths for Sr-HT and HA particles. (b) Displays the results of tensile and shear tests for Sr-HT coatings, demonstrating the mechanical adhesion and integrity of the coatings. (c) Provides a comparative analysis of the surface morphology and antibacterial activity of Sr-HT and HA coatings against MRSA (Gram-positive) and *P. aeruginosa* (Gram-negative) bacteria, with fluorescent images indicating bacterial adhesion and viability. (Adapted with permission from Ref. [152].

Strontium, which is essential for the development of bioceramic applications involving bone regeneration and implant integration, is renowned for its osteoinductive and antibacterial properties. Substantial antibacterial activity has been observed in Sr-doped hardystonite (Sr-HT), an exceptional coating, against formidable pathogens including *Pseudomonas aeruginosa* and MRSA, strains notorious for their resistance to antibiotics (Fig. 6) [152]. By enhancing its antibacterial properties and stimulating bone repair and regeneration, hardystonite becomes a valuable material for dental and orthopaedic implants when Sr is infused into it. Technological advancements encompass Sr-doped chromium oxide, which is renowned for its effectiveness against multidrug-resistant *Escherichia coli* [153]. The application of Sr and selenium co-substituted HA in bone tissue engineering is enhanced by the combination of the antimicrobial and bone-regenerative properties of both elements [154]. Moreover, due to their increased surface area and irregularity, Sr-doped titanium dioxide nanorods show enhanced cellular responses and heightened antibacterial activity [155,156]. Finally, amorphous Sr-doped calcium phosphate is identified as a substance capable of remineralising dental structures and enhancing resistance to cariogenic bacteria [157]. A multifaceted strategy is exemplified by the incorporation of Sr into diverse bioceramics, which provides improved mechanical, chemical, and biological characteristics that are essential for medicinal applications.

#### 4.1.4 Antibacterial activity of zinc ions

Zn^2+^ interact with the negative charge of the bacterial membrane. A strong ionic connection between Zn^2+^ and the bacterial surface is created by reverse charges, causing electrostatic forces. Furthermore, binding Zn^2+^ to the bacterial membrane may increase membrane permeability, causing pores in the bacterial surface, and triggering membrane rupture and cytoplasmic leaking, resulting in cell death [158]. Zn^2+^ has a role in regulating bacterial cell proliferation, differentiation, and membrane structure conservation. Additionally, they participate as cofactors in many critical metabolic pathways, including the synthesis and breakdown of carbohydrates, lipids, and proteins [159,160]. The positive effects of Zn are dominant at low concentrations, whereas high amounts impede bacterial development. For example, an excess of Zn^2+^ may compete with other metals and induce a metal mismatch in various metal-binding proteins, resulting in protein dysfunction, enzymatic inactivation, or protein denaturation [161].

#### 4.1.5 Antibacterial activity of iron ions

Iron (Fe) is a critical microelement for bacterial life and is involved in various biological processes, including DNA synthesis and energy metabolism. On the other hand, iron can be toxic to bacterial cells in high concentrations. In the biology process, iron is found in two oxidation states: oxidised Fe^3+^ and reduced Fe^2+^. Although bacteria can acquire Fe^3+^ from their environment, they rapidly convert it to Fe^2+^. In addition, Fe^2+^ produces a significant amount of hydroxyl radical (^•^OH) by speeding the Fenton and Haber-Weiss reactions [162]. These radicals contribute to oxidising the lipids of the membrane and damaging the proteins and DNA. In addition, free radicals produced in this way, such as superoxide ion (O_2_^•^^−^ ) and ^•^OH, can rupture the cell envelope via a electrostatic, dipole-dipole, hydrogen bond, hydrophobic, and van der Waals forces. This results in the disarray and destruction of the cell membrane, resulting in the death of the bacterium [163].

#### 4.1.6 Antibacterial activity of gallium ions

Gallium (Ga) is a semimetal element that stimulates osteoblasts and promotes bone growth [164]. Gallium ions of Ga^3+^ may substitute for Fe^3+^ ions in many metabolic reactions due to their high resemblance (i.e., same ionic radius, electronegativity, coordination number, etc.). With this capacity, gallium serves as a diagnostic and therapeutic agent for metabolic problems of both soft and hard tissues. Recent research has also shown that gallium ions have an antibacterial effect based on the exchange of iron ions in the process of protein metabolism. Ga is a group III transition metal that exhibits promising antibacterial activity against various pathogens. Ga ions can interfere with bacterial metabolism, DNA replication, and other critical cellular processes, ultimately leading to bacterial cell death. Ga also exhibits synergistic effects with other antibiotics, enhancing their antibacterial activity and reducing the development of antibiotic resistance. Moreover, Ga-based materials, such as Ga nanoparticles, can be synthesised and modified for various biomedical applications, including wound healing, implant coatings, and drug delivery systems. Despite the promising results, further studies are needed to investigate the antibacterial mechanisms of Ga and optimise its efficacy for clinical use.

**Fig. 7:**
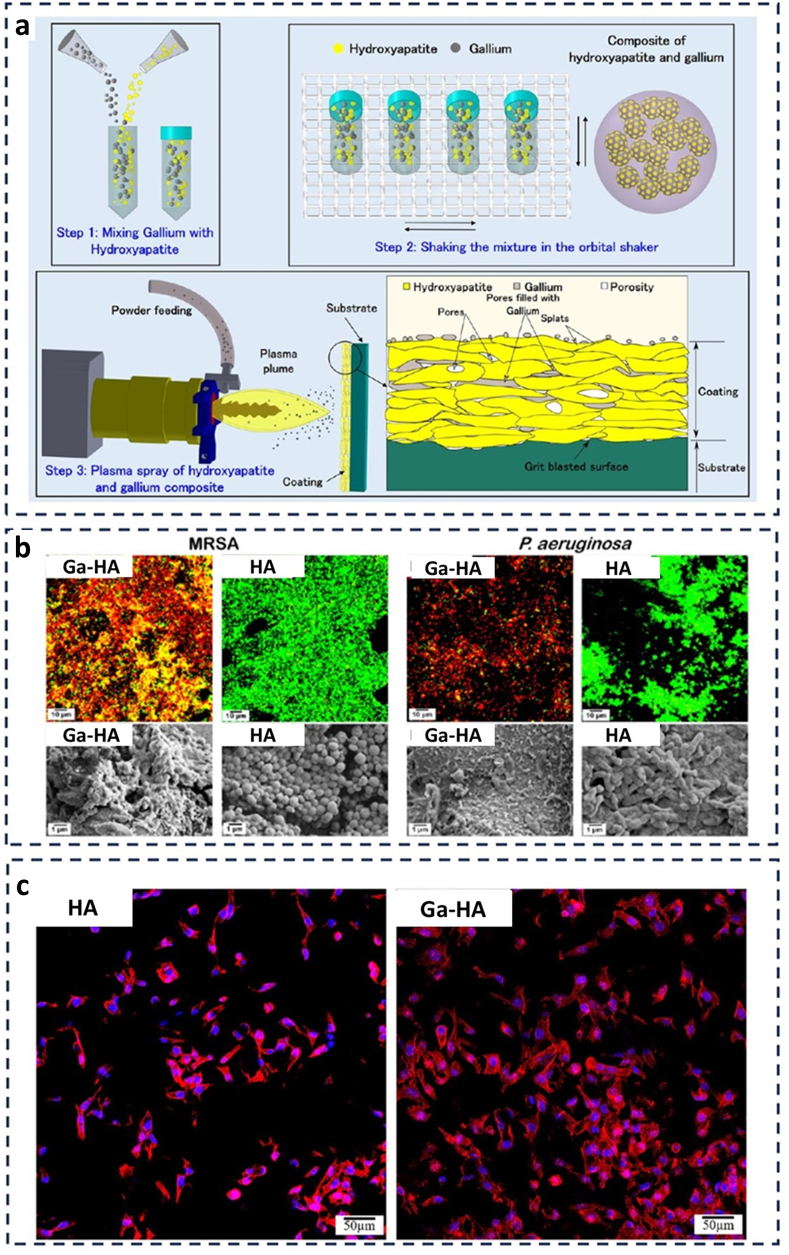
Synthesis and antibacterial evaluation of Ga-HA. (a) Depicts the sequential procedure for fabricating a composite material of HA and gallium. This involves the initial stage of blending the components, followed by agitation in an orbital shaker, and ultimately applying the mixture onto a substrate using plasma spraying. (b) Illustrates the antimicrobial properties of Ga-HA against MRSA and *P. aeruginosa*. It includes confocal microscopy pictures that display live/dead staining, as well as scanning electron microscopy images that disclose the surface morphology of the coatings. (c) Demonstrates the interaction between HA and Ga-HA with cellular structures, emphasising the ability of the doped material to support bone growth. This is shown using fluorescent labeling to visualise the cell nuclei and actin filaments (Adapted with permission from Ref. [45]).

Gallium-doped HA (Ga-HA) is a biomaterial that is increasingly being used in antibacterial orthopaedic applications (Fig. 7) [45]. By introducing gallium ions into HA matrices, the biocompatibility and osteoconductivity of HA are maintained while simultaneously providing strong antibacterial effects. Ga-HA has exhibited a notable capacity to impede the growth and reproduction of several harmful bacteria, such as MRSA and *Pseudomonas aeruginosa*, which are frequently responsible for implant-associated illnesses. The antibacterial effect of Ga is due to its capacity to break the integrity of bacterial cell walls and hinder essential metabolic pathways. Furthermore, the ability of Ga to imitate calcium interferes with the iron metabolism in bacteria, enhancing its antibacterial effectiveness. The dual activity of Ga-HA renders it highly suitable for bone tissue engineering applications that require both infection prevention and bone regeneration. This composite material utilises a synergistic approach by combining the proven ability of HA to regenerate bone with the effective antibacterial properties of Ga. This creates new opportunities for the advancement of biomedical implants.

### 4.2 Metal and ion doping in bioceramics: a strategic approach to antibacterial efficacy

**Fig. 8:**
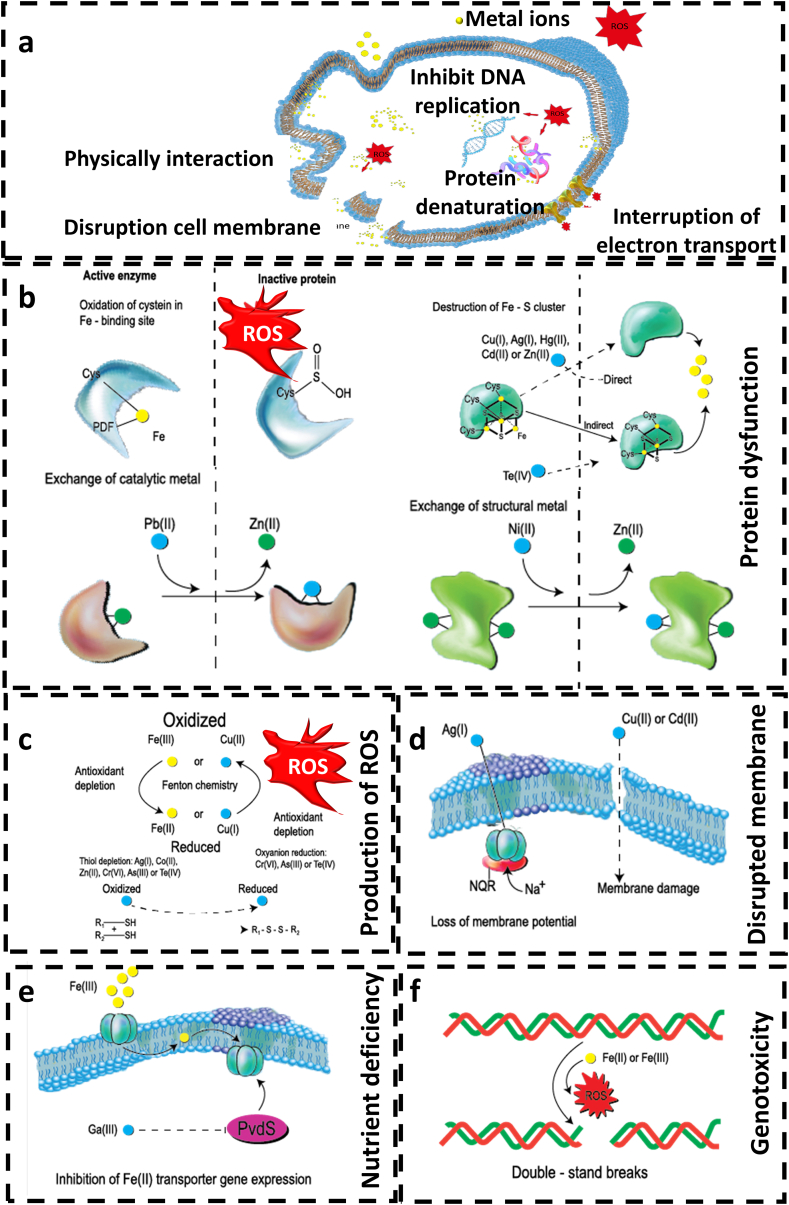
Antibacterial mechanisms involve interconnected processes such as protein dysfunction, oxidative stress, membrane impairment, nutrient interference, and genotoxic effects. (a) Antibacterial activity is multiple and often interconnected mechanisms. (b) Metals and ions toxicity can cause protein dysfunction by inhibiting enzyme activity. (c) The formation of extracellular and intracellular reactive oxygen species (ROS) and the depletion of antioxidants result in oxidative stress and the damage of lipids, proteins, and DNA. (d) Metals and ions interact with the cell membrane via electrostatic interactions, impairing membrane function. (e) The toxicity of some metals and ions can interfere with nutrient assimilation. (f) Metals and metal ions can be genotoxic, interfering directly with proteins and DNA, impairing their function and disrupting cellular metabolism.

Understanding the antibacterial mechanisms of metals and metal ions is crucial for optimising their performance in clinical applications. In this section, we discuss four primary mechanisms of antibacterial properties associated with metals and metal ions: (1) generation of reactive oxide species, (2) protein dysfunction and loss of enzyme activity, (3) disruption of membrane function, and (4) genotoxicity. Fig. 8 illustrates these mechanisms, providing a comprehensive overview of how metals and metal ions exert their antibacterial effects on bacteria.

#### 4.2.1 Generation of reactive oxide species

Some metal ions have been demonstrated to induce intracellular ROS in various studies. For example, exogenous hydrogen peroxide or substances that catalyse the generation of O_2_^•^^−^, such as paraquat, cause DNA damage and block some enzyme activity required for cell development in *E. coli* [165,166]. Mutants lacking in ROS-scavenging enzymes and other cellular antioxidants commonly show altered sensitivity to Cr, As, Te, Fe, and Cu [166]. Cu(II) or Te(IV) exposure reduces enzyme activity, causing DNA damage. Thus, metal toxicity may be owing to ROS-mediated cellular damage, and different metal-catalysed oxidation processes may cause specific protein, membrane, or DNA damage [166]. The mechanisms that have been proposed to account for the increased ROS production include:

First, redox-active transition metals other than Fe, such as Cu, Cr, Co, V, and Ni, can catalyse Fenton chemistry [167]. Cu has been demonstrated to catalyse the formation of hydroxyl radicals, and other metals may do the same [168]. Because numerous variables influence the ability of these transition metals to engage in Fenton chemistry, determining whether these reactions occur *in vivo* and their rates is difficult [168]. On the other hand, in phosphate buffer at pH 7, Cu(I), Cr(I), and Co(I) catalyse Fenton chemistry faster than Fe(I), whereas Ni(I) catalyses this reaction at a slower rate [166,169].

Second, transition metals may interfere with the coordination of Fe. The solvent-exposed [4Fe–4S] clusters of proteins appear to be the principal metal targets. However, most of these atoms are sheltered by coordination bonds, and only 20 % are projected to be Fenton active. Metals can directly or indirectly destroy [4Fe–4S] clusters, releasing Fenton-active Fe into the cytoplasm and increasing ROS production [170]. Some Fenton-inactive metals (including Ag, Hg, and Ga) create ROS, which may explain why bacteria require or upregulate ROS-detoxification enzymes to tolerate hazardous concentrations of these elements [166,171].

Third, the thiol-mediated reduction of various metal species, such as Fe(III) and Cu(III), produces ROS via intermediate radical chemistry [172]. Moreover, thiol-mediated reduction can create Fenton active metal species such as Cr(III), Cr(IV), and Cr(V) [173]. Fe(III), Cu(II), and Cr(VI) result in the generation of reactive oxygen species (ROS) via a sulphur radical intermediary. Reduced thiols, such as glutathione (GSH), are a critical antioxidant in the bacterial cell. GSH, on the other hand, can be depleted by oxidising thiophilic metals such as Ag, cadmium (Cd)(II), or (As) (III). Thus, the anti-oxidative defences of bacteria are weakened, and its vulnerability to subsequent metal-mediated ROS increases [174]. However, whether radicals are involved in ROS generation or microbial metal poisoning *in vivo* is unclear.

#### 4.2.2 Metals and ions causing protein dysfunction and loss of enzyme activity

Recent research revealed that intracellular proteins are also targets of metal toxicity due to the abundance of amino acid-mediated binding sites, which primarily consist of reduced thiols from cysteine side chains, carboxy groups from aspartates and glutamates, and highly reactive primary amines from lysine side chains [174]. When metal ions bind to susceptible amino acids, they catalyse their oxidation, impair protein function, decrease protein stability, and mark the protein for degradation [175]. Another study showed that Gallium ions inhibit or kill bacteria by taking up another metal ion instead of the essential one due to their chemical similarities. Once inside the cell, these ions disrupt metabolic pathways due to the inability of bacterial cells to reduce them, irreversibly impairing cell metabolism [176].

Some metals and ions can potentially cause site-specific damage to biological proteins. For example, hazardous concentrations of Cr(vi) rapidly increase protein carbonyl levels in *S. cerevisiae* within minutes [177]. In *E. coli*, just a few amino acid residues per protein are vulnerable to metal-catalysed oxidation [178]. Metal-catalysed oxidation of amino acid side chains produces carbonyl compounds employed as a marker of oxidative protein damage. *In vivo*, oxidation of amino acid side chains can reduce catalytic activity and cause protein degradation. Thus, metals could potentially cause site-specific damage to biological proteins, leading to metal poisoning. The most heavily oxidised proteins are cytosolic enzymes involved in glycolysis or subsequent catabolic reactions. A bacterial Fe–S dehydratase family is susceptible to metal-specific inactivation [166]. These elements harm Fe–S–containing dehydratases *in vitro* and inhibit them *in vivo* at doses that cause bacteriostasis [170]. This suggests the metals inflict little or no further damage to the proteins compared to proteins not repaired by proteins like cysteine desulphurase or SufA. However, in this case, the destruction of the [4Fe–4S] clusters in dehydratases occurs exclusively in aerobic conditions. Unlike soft metal cations, the metalloid oxyanion Te(iv) promotes indirect oxidation of Fe–S clusters, most likely via ROS intermediates [166].

#### 4.2.3 Metals and metal ions induce the function of membranes

Metal cations adsorb on polymers with strongly electronegative chemical groups in bacterial membranes and exert bactericidal toxicity [179]. Exposure to toxic metals such as Ag and Al dramatically alters the cytoplasmic membrane integrity of *E. coli and S. aureus.* Cell death can be attributed to membrane disruption or apparent cell wall separation. Other research suggests that certain metals, particularly Ag, harm the bacterial electron transport chain. Lipid peroxidation has also been connected to the toxicity of Cu(II) and Cd(II) in bacteria and yeast, and it has been hypothesised that this is a deadly mode of action for antimicrobial metallic surfaces constructed of Cu and its alloys. This is consistent with the discovery that metal exposure increases the concentration of thiobarbituric acid reactive compounds in cell extracts. Consistent with this concept, introducing genetic alterations that enhance the unsaturated fatty acid content of cell membranes or feeding the growth medium with polyunsaturated fats leads to an increase in the level of thiobarbituric acid reactive substance after metal exposure.

#### 4.2.4 Genotoxicity

Genotoxicity is a term that relates to the use of mutagens such as Cr (IV) and other cations that damage DNA [180]. For example, many studies have indicated that when *E. coli* is exposed to high quantities of iron, the bacteria DNA damage can be catalysed by Fe-mediated Fenton chemistry, resulting in lethal DNA damage [181]. Increased Fe concentrations in bacterial cells are caused by mutations that cause Fenton-active Fe to accumulate in the cell, which accelerates DNA damage and results in cell death [182].

Metals and metal ions in high concentrations are toxic to prokaryotic cells due to their redox properties. For example, numerous studies have linked the antibacterial activity of Cu to its ability to transition between Cu^+^ and Cu^2+^, which can generate reactive oxygen species under aerobic conditions. Copper Fenton explains how H_2_O_2_ decomposes into ^•^OH, leaving the catalytic metal in its oxidised state. Additionally, Cu has been associated with extracellular DNA damage during cell lysis, which may limit post-mortem horizontal gene transfer of resistance via transformation [180]. However, Cu^2+^ preferred oxidation state in solution. As a result, a reducing agent (O_2_^•−^, NADPH oxidase from the respiratory chain, or intracellular thiols) is required to convert Cu^2+^ to Cu^+^ and continue the production of ^•^OH. Warnes et al. showed that Cu toxicity for *Enterococcus faecalus* and *Enterococcus faecium* involves direct and indirect copper ion action, ROS production, and respiratory chain and DNA repair failure. The authors contend that Fenton production of ^•^OH is not the fundamental cause of DNA damage, but rather Cu (II)-induced denaturation of bacterial DNA. The same authors later validated the function of ROS in methicillin-resistant *S. aureus* DNA damage [183].

### 4.3 Engineering surface topography for anti-biofouling: lessons for designing bioceramics

Surface topography is typically altered through the use of anti-biofouling techniques that result in passive structuration [184]. As bacteria come into contact with the surface structure, these strategies entail employing structural units such as polymer brushes or nanotubes placed over the surface, preventing them from adhering and breaking apart the membrane as they come into contact with it. These approaches increase antifouling activity over longer periods, although they may not effectively eradicate an infection [88]. Additionally, surface alterations can have a broader biological impact. In addition to influencing bacterial adherence, the final topography of glass surfaces with various nanostructures affects bacteria metabolism [185,186].

Natural antibacterial activity based on mechanical interactions was first reported. For example, the wings of insects such as cicadas or dragonflies are nanopatterned with high-aspect-ratio cone-like nanopillars that are toxic to bacteria such as *P. aeruginosa* [187]. This may result from an evolutionary response to the environment, which prevents the production of biofilms that impair the aerodynamics of such insects.

Studying bacterial adhesion to bioceramics is crucial for understanding and improving antibacterial activity because it provides insights into the initial stages of bacterial colonisation on implant surfaces. Bacterial adhesion is the first step in developing biofilms, which are complex communities of microorganisms embedded in a self-produced extracellular matrix. Biofilms are highly resistant to antibiotics and the host immune system, making them challenging to treat and eradicate. By studying bacterial adhesion to bioceramics, we can identify factors that influence this process and develop strategies to minimise bacterial colonisation. This could include modifying the surface properties of bioceramics or incorporating antibacterial agents into their composition. Understanding bacterial adhesion to bioceramics is essential for designing next-generation implant materials with enhanced antibacterial properties, ultimately reducing the risk of implant-associated infections and improving patient outcomes.

#### 4.3.1 Surface properties influencing bacterial adhesion

The chemical composition of the surface influences the microorganisms attached to it [188]. Because surface chemistry determines the ability of bacteria to adhere, maintaining a smooth substrate is crucial [189]. Additionally, bacterial adhesion was shown to be greatest on hydrophilic surfaces with positive surface charge characteristics, followed by hydrophobic substrates with negative surface charge characteristics, and lowest on hydrophilic substrates with negative surface charge characteristics [190]. The extremely hydrophobic or extremely hydrophilic surfaces inhibited *E. coli* adherence [191]. This finding contradicts normal surface chemistry adhesion trends for hydrophilic bacteria, which show that bacterial adherence increases with increasing hydrophobicity and decreases with decreasing surface energy. The conflict could be caused by surface roughness and topography, which affect bacterial adhesion behaviour when surfaces interact with bacteria.

**Fig. 9:**
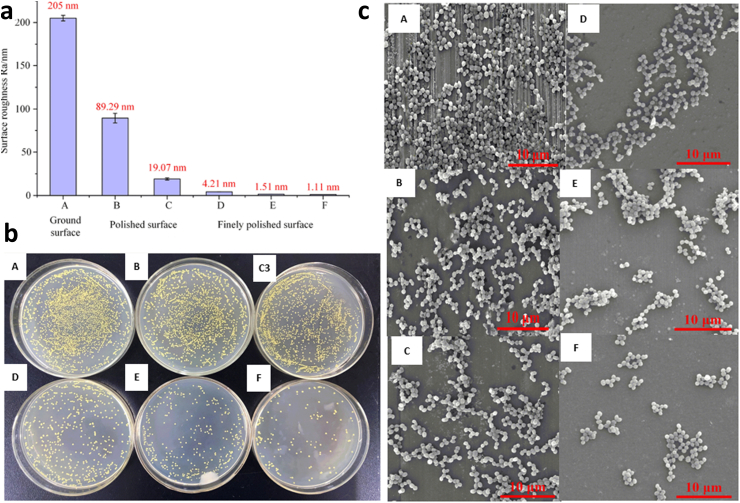
The morphology and distribution of the attached *S. aureus* bacteria on the state variation from rough to smooth of Y-TZP bio-ceramic surfaces. (a) The average surface roughness with A (205 nm), B (89.29 nm), C (19.07 nm), D (4.21 nm), E (1.51 nm), F (1.11 nm). (b) The CFU results show the number of bacteria adhered to the sample surfaces. Sample A has the largest number of adhering bacteria, samples B–E, the number of adhering bacteria gradually decreases, and sample F has the least number of adhering bacteria on the surface. (c) The morphology and density of the bacteria adhering to the surface under SEM. Microorganisms almost totally cover the surface (Ra 205 nm). The protruded topographical characteristics of the surface layer (R_a_ 89.29 nm) are removed, and the places where the bacteria can be attached and fixed are less. The smooth region of the surface (Ra 19.07 nm) gradually grows as the original ground textures and flaws are removed. The surfaces (Ra 4.21 nm and 1.51 nm) the number and density of the bacteria adhering to the surface are reduced. The quantity and density of the adhering bacteria are the lowest because no texture remains on the surface, and the entire surface becomes exceedingly smooth (Adapted with permission from Ref. [198]).

A rough surface has more surface area, which makes it more favourable for bacteria to adhere [192]. However, the influence of surface roughness on adhesion appears to be related to the degree of roughness, surface topography, and material compositions [193]. Some previous research has been conducted on bioceramic materials; however, the findings remain contentious. Wassmann et al. studied the adherence of *S. epidermidis* to the surface of a zirconia ceramic implant [194]. The findings demonstrate that altering the roughness of the surface has no influence on the quantity of bacterial adherence. Dutra et al. investigated the influence of surface roughness on bacterial adherence to zirconia-stabilized yttria (Y-TZP) ceramics [195]. The authors discovered that the quantity of adhering bacteria remains constant when the surface roughness is reduced. Thus, surface roughness does not play a significant role in bacterial adherence, and a Y-TZP material exhibits a low susceptibility to bacterial adhesion. Kang et al. investigated the effect of several polishing procedures on the roughness and adherence of *Streptococcus mitis* on zirconia ceramic surfaces [196]. The results indicate that the quantity of adhering bacteria increases proportionately when the surface roughness rises. The previous studies on the sample surfaces have employed roughness values in the micron to the submicron range. A bioceramic surface with a roughness of nanoscale or submicron is unknown regarding bacterial adherence. The surface topography influences bacterial adherence, as shown in Fig. 9. Because *S. aureus* has low mobility and prefers low-lying topographies like valleys, grooves, and pits, a rough, rugged, high-amplitude groove-like surface topography significantly promotes initial *S. aureus* adhesion and biofilm growth [197,198]. Defined micro-scale features such as scratches, pits, and grooves will boost the bacterial-surface attachment strength [199]. In Fig. 9, for example, samples A, B, and C have more bacterial adhesions than samples D, E, and F because they contain more micro-scale characteristics. Sample E (R_a_ 1.51 nm), with a shallow unidirectional surface texture, has more sites for initial bacterial attachment than sample F (Ra 1.11 nm), with a homogeneous and damage-free surface, resulting in higher bacterial adherence.

Notably, there have been inconsistent findings addressing the bacterial adhesion patterns in relation to surface chemistry due to a variety of factors. Firstly, the hydrophobicity of the substrate, the roughness and texture of the substrate, as well as the porosity and fibrousness of the material, can all affect bacterial adhesion behaviour [190,[200], [201], [202]]. Limiting the substrate roughness effect is necessary to deduce precise correlations between substrate chemistry and bacterial adherence. Second, variations in experimental assays used to determine bacterial adhesion, such as drop-casting inoculation and the rinse phase, have been observed due to velocity gradient, gravity, and drying effects [190,203,204].

Achieving the balance between surface characteristics that inhibit bacterial adhesion while simultaneously promoting osteogenic cell functions represents a pivotal challenge in designing advanced bioceramics for orthopaedic applications. This balance is essential to prevent infection and ensure the successful integration of the implants with bone tissue. Current research efforts focus on engineering bioceramic surfaces with dual functionality. One effective strategy involves crafting a moderately rough surface topography incorporating precisely defined micro- and nano-scale features. These features are strategically designed to reduce bacterial attachment by minimising areas where bacteria can easily adhere while providing the necessary cues to enhance osteoblast adhesion, proliferation, and differentiation. This is achieved through careful modulation of the mechanical strength of surface and its chemical and physical properties.

Furthermore, the quality of implant surfaces is defined by an intricate interplay of mechanical, topographical, and physicochemical properties. Alterations in one property can significantly impact the others, influencing the overall biological response to the implant [205]. For example, increasing the micro-scale roughness might enhance bone cell functions. Still, it could potentially also provide more niches for bacterial growth unless nano-scale modifications are simultaneously applied to counteract this effect. As a result, the physical and chemical properties of bioceramics can be tailored with high precision to achieve these multifaceted requirements. Advanced manufacturing techniques such as laser micromachining and chemical vapour deposition are employed to fine-tune these properties, allowing for the development of surfaces optimally designed to combat bacterial colonisation while supporting osteogenesis.

#### 4.3.2 Environmental factors influencing bacterial adhesion

Environmental factors that influence bacterial adherence include temperature, bacterial density, chemical modification, antimicrobial existence, and associated flow characteristics [206]. Flow parameters are considered dominating elements because they have a major impact on the number of bacteria adhered to as well as the structure and function of the biofilm. Katsikogianni et al. demonstrated that flow conditions affected bacterial adherence to various substrates. The quantity of adhering bacteria reduced dramatically as the shear rate was increased from 150 sec^−1^ to 1500 sec^−1^ [207]. Mohamed et al. demonstrated that when the number of receptors per cell is increased, *S. aureus* adherence to collagen-coated coverslips increases between 50 and 300 sec^−1^ and decreases above 500 sec^−1^ [208]. Therefore, bacterial attachment does not have an optimal flow rate because the delivery rate must be matched with the force acting on the enclosed bacteria.

Adhesion can be affected by changes in pH and electrolyte concentrations of KCl and NaCl [209]. Bacteria adjust their activity and synthesis of proteins involved in various cellular activities in response to changes in internal and external pH. Studies have demonstrated that a gradual increase in acidity is preferable to a quick increase caused by adding HCl [210]. This indicates that bacteria have systems in place that enable them to adjust to tiny changes in the pH of their surroundings. However, other biological functions do not adapt as readily to pH variations. The pH of the bone tissue environment is frequently below 7, whereas the pH of healthy tissues is typically in the range of 7.35–7.45, depending on the tissue [211]. HA and biphasic calcium phosphate (BCP) are two ceramic materials commonly used as bone substitutes. The porosity of these materials, as well as the decrease in pH in the surrounding area as a result of surgical trauma, may, however, predispose them to bacterial infections. Recent studies demonstrated that when the pH of the solution was reduced from 7.4 to 6.8, the adhesion of S*taphylococci* to both HA and BCP surfaces was dramatically reduced [210,211]. Furthermore, they discovered in this investigation that the pores in HA and BCP ceramics were not large enough to allow the internalisation of *Staphylococci*. As a result, their anti-adherent capabilities appeared to improve when the pH value was dropped, indicating that the bioceramics HA and BCP are not impaired when used in orthopaedic applications. In addition, using tricalcium silicate-based cement such as MTA, a previous study demonstrated that the pH of MTA is 10.5 at the time of mixing and can increase to 12.9 after 3 h of setting [210]. High pH affects the structure of endodontic bacterial cells by producing DNA breakdown and cellular protein damage, which results in a reduction in the survival of the bacterial cells. MTA has antibiofilm action; the pH increase during the setting process is primarily responsible for this. The generation of calcium hydroxide during the hydration reaction of MTA has the potential to cause the pH to rise. Therefore, MTA demonstrated antimicrobial action against a variety of different bacterial biofilms, including those formed by *Staphylococcus aureus*, *Escherichia coli*, *P. aeruginosa*, *Porphyromonas gingivalis*, and *Candia albicans* [212,213].

#### 4.3.3 Proteins corona influencing bacterial adhesion

Biomolecules such as extracellular matrix proteins adsorb onto the material surface when an implant is inserted into host tissue, generating a conditioned protein coating that promotes adherence of free-floating planktonic bacteria to the implant surface. Then, the adhering bacteria divide the number rapidly and release adhesive secretions, creating dense colonies of connected cells called biofilms [214,215]. Notably, serum or tissue proteins such as albumin, fibrinogen, laminin, and denatured collagen enhance or inhibit bacterial adherence. These proteins attach to the surface of the substratum, adhering to the bacterial surface or remaining present in the liquid medium during the adhesion period. Most interactions between bacteria and proteins occur through specialised ligand-receptor interactions rather than broad bindings. Proteins can alter the adhesion behaviour of bacteria by modifying the physicochemical properties of the surface membrane of bacteria [216,217].

Fibronectin is a protein that increases adhesion-promoting bacterial adherence to biomaterial surfaces [218] especially S*taphylococci* [219]. There are two known S*taphylococci* binding sites in Fn: one in the N-terminal domain and the other towards the C-terminus. *S. epidermidis* has a greater affinity for the C-terminal segment and many bacteria have fibronectin-binding proteins that can bind to an increasing number of Fn sites [220,221]. *S. aureus* generates surface proteins that may be important in initial host tissue attachment. The microbial surface components recognising adhesive matrix molecules bind specifically to extracellular matrix components. *S. aureus* has two FnBPs, FnBPA and FnBPB, encoded by the closely related genes *fnbA* and *fnbB*. Almost all clinical isolates of *S. aureus* have one of two genes. FnBPs are implicated in infection pathogenesis. Almost all clinical isolates of *S. aureus* have the surface proteins FnBPA and FnBPB. FnBPs are involved in the pathophysiology of infection [221].

Bacterial adherence to biomaterials and host tissues is also mediated by fibrinogen. Fibrinogen increases bacterial adherence by connecting biomaterial surfaces with fibrinogen membrane receptors [222]. *In vivo*, bacteria that can specifically bind surface-adsorbed fibrinogen are reported to be more clinical orthopaedic device-associated infections [223]. Charville et al. found that *S. aureus, S. epidermidis*, and *Escherichia coli* adhered better to pre-adsorbed fibrinogen than to protein-free PVC substrates [224]. The greatest increase was seen in *S. aureus*, whose adherence to fibrinogen-coated substrates was five times that of uncoated controls. *S. aureus* adhesion to polyurethane surfaces with pre-adsorbed fibrinogen was reported by Baumgartner et al. Pei et al. showed that *S. epidermidis* adhesion to non-functional fibrinogen-coated control catheters was roughly half that of fibrinogen-coated catheters [225]. The observations suggest that certain fibrinogen-mediated interactions between bacterial cells and substrates improve bacterial adherence in the presence of fibrinogen.

#### 4.3.4 Hydrogels with surface-modified bioceramics for infectious bone repair

Hydrogels and surface-modified bioceramics combined constitute a potentially effective strategy for tackling the difficulty associated with infectious bone repair [226]. Combining two complementary components can create multifunctional bioceramics with enhanced antimicrobial properties and improved bone regenerative capabilities. The ability of hydrogels to mimic the extracellular matrix can serve as effective carriers for the delivery of antimicrobial agents, such as antibiotics [227], antimicrobial peptides [228], or metal ions [229]. The hydrogel matrix can facilitate the controlled and localised release of these agents, helping to combat bacterial colonisation and biofilm formation at the site of infection. Furthermore, the hydrogel component can improve the handling properties and drug-loading capacity of the bioceramic scaffolds [230], overcoming some of the limitations associated with direct bioceramic implantation.

### 4.4 Smart antibacterial bioceramics with stimulus-responsive mechanisms: emerging innovations for medical implant applications

Smart antibacterial bioceramics with stimulus-responsive mechanisms offer a promising and innovative way to address implant-related infections. Despite being in early development stages, these bioceramics have piqued the interest of the research community due to their potential to transform medical implant technology. However, there is a limited amount of studies on smart antibacterial bioceramics, making this research a valuable addition to the field.

Antibacterial stimuli-responsive bioceramics are designed to release antimicrobial agents in response to environmental cues such as temperature, pH, or the presence of bacteria [231]. This targeted release allows for more efficient and effective bacterial eradication while minimising the risk of antimicrobial resistance and adverse effects on surrounding healthy tissues. To optimise the performance of these smart materials, a comprehensive understanding of the interactions between the bioceramic matrix, antimicrobial agents, and environmental stimuli is necessary.

Future research will focus on optimising the response time and sensitivity of smart antibacterial bioceramics to specific environmental cues, as well as ensuring their long-term stability and biocompatibility. These include incorporating smart polymers that respond to environmental changes, designing bioceramics with switchable surface properties to control bacterial adhesion and release, and creating coatings or surface treatments to detect and respond to particular bacterial species or infection biomarkers.

To fully realise the potential of stimulus-responsive antibacterial bioceramics, many obstacles must be addressed despite the growing interest in this field. These obstacles include optimising the response time and sensitivity of the materials to specific stimuli, ensuring long-term stability and biocompatibility, and scaling up manufacturing processes for clinical use. In addition, additional *in vitro* and *in vivo* studies are required to evaluate the safety, efficacy, and overall performance of these materials in various medical applications.

In conclusion, smart antibacterial bioceramics with stimulus-responsive mechanisms represent an exciting and emerging research field with the potential to impact implantable biomaterials substantially. By addressing current challenges and advancing our understanding of these materials, researchers can create innovative solutions for infection prevention and treatment in medical implant applications, thereby improving patient outcomes and reducing healthcare costs.

## 5 Challenges and future directions

Key points:

• Developing complex antibacterial bioceramics requires a careful balance between innovation and practicality due to challenges in manufacturing, regulation, and clinical integration.
• Antibacterial bioceramics are being extended from bone tissue engineering to other areas like ophthalmology, showcasing their broad potential in improving patient care.

Developing bioceramics with enhanced antibacterial properties is critical to addressing the growing problem of implant-associated infections. However, the complexity of these bioceramics frequently creates significant challenges to their successful clinical translation [232]. Complex bioceramics, while often demonstrating superior antibacterial properties, can be challenging to manufacture on a large scale [233]. Incorporating multiple functions, such as customised surface topographies, controlled release of antimicrobial agents, and intrinsic antibacterial mechanisms, can greatly complicate the manufacturing process. This complexity can result in greater production costs, lower scalability, and challenges in promising consistent quality and accuracy, critical for successful clinical adoption. Moreover, incorporating these complex biomaterials into current clinical processes can be a challenging endeavour, demanding significant changes to established methods and practices [234]. Regulatory organisations frequently need significant testing and documentation to assure the safety, efficacy, and reliability of these complex biomaterials, which might cause delays in their clinical implementation [234].

In contrast, simpler bioceramics are more likely to be adopted successfully in clinical applications [234]. Due to the simplicity of their production, they are more readily available and cost-effective. Regulatory agencies are more inclined to approve less complex bioceramics due to the ease with which their safety and effectiveness can be evaluated. Furthermore, integrating less complex bioceramics into established clinical workflows frequently results in insignificant disruption. Despite these challenges, developing improved antibacterial bioceramics is critical. The complexity of these materials must be carefully balanced with practical clinical translation considerations. Strategies that prioritise the balance of antibacterial efficacy and manufacturing simplicity, regulatory compliance, and clinical integration may be critical to successfully bringing these next-generation biomaterials to the centre of patient care.

The traditional focus on antibacterial bioceramics in bone tissue engineering is now being expanded in various tissue interfaces, demonstrating the broad applicability and versatility. Notably, recent advancements in research introduce antibacterial bioceramics into ophthalmology, a significant shift from their conventional use. The development of antibacterial glass-based sputtered coatings for ocular prostheses offers a novel approach to reducing infection risks associated with ocular implants [235]. This innovation highlights the adaptability of bioceramics to diverse biological environments and their potential to transform ocular prosthetic applications by enhancing safety and efficacy. Additionally, exploring bioactive glass and glass-ceramic for orbital implants further emphasises the expanding scope of bioceramic applications beyond skeletal repair towards improving outcomes in ocular rehabilitation [236]. These emerging applications highlight a pivotal evolution in the use of antibacterial bioceramics, from their foundational role in bone regeneration to their promising potential in addressing complex challenges in ophthalmic medicine. The versatility and adaptability of bioceramics to various tissue interfaces offer great potential for enhancing patient care and clinical outcomes in numerous medical disciplines.

## 6 Conclusions

To tackle the increasing worldwide risks posed by drug-resistant pathogenic microbes, researchers have made significant efforts to create antimicrobial bioceramics that not only kill pathogenic microbes but also promote the adhesion and growth of healthy cells. This means the next-generation bioceramics for biomedical applications should simultaneously prevent microbial infection and promote tissue regeneration. Perfect bioceramics should have superior biocompatibility and antibacterial properties to suppress bacterial growth and secondary infections, as well as sufficient mechanical resistance for surgical insertion. Bioceramics have been promising biomaterials for various biomedical applications, such as orthopaedic implants and tissue regeneration, for a long time. Bioceramics with metal ions have been successfully doped in recent research as prospective therapeutic agents for improved bone regeneration. The combinatory features of ion-doped bioceramics led to an increased interest in these materials for various biomedical applications including bone regeneration. Due to the continuous emergence of bacterial resistance, an increasing amount of research is devoted to developing novel antimicrobial agents. Antibiotic-resistant bacterial strains pose an increasing threat, necessitating the development of effective and long-lasting antibacterial materials. Metals have been used, and their antimicrobial properties have been extensively studied. Similar to antibiotics, metals have a distinct effect on bacterial and mammalian targets due to their divergent metal transport systems and metalloproteins. This enables the long-term use of metal-based bioceramics as antimicrobial agents with minimal adverse effects on the host. Identify the qualities of surfaces that bacteria feel, decipher the molecular mechanisms by which bacteria sense surfaces, and identify how to adjust surfaces. Microbes interacting with surfaces remain a mystery in physics, biochemistry, genetics, biomedical, and biotechnology. The chemical composition of the substance, the surface charge, the hydrophobicity, and simply the surface roughness or physical configuration all influence the adhesion of bacteria to a biomaterial surface. Additionally, their surface energy, the number of unoccupied binding sites, and hydrophobic/hydrophilic properties can be rapidly altered by serum protein adsorption or binding and the production of biofilms. Antibacterial properties of bioceramics prevent bacteria from adhering to implant surfaces, minimising infection risk. Multifunctional bioceramics offer many advantages and the capacity to fine-tune them, making them an indispensable component in biomedical applications. As discussed in this review, new approaches have the potential to understand bacteria–surface interactions and guide applications for orthopaedic implants.

## CRediT authorship contribution statement

**Ngoc Huu Nguyen:** Writing – review & editing, Writing – original draft, Visualization, Conceptualization. **Zufu Lu:** Writing – review & editing, Conceptualization. **Aaron Elbourne:** Writing – review & editing. **Krasimir Vasilev:** Writing – review & editing, Conceptualization. **Iman Roohani:** Writing – review & editing. **Hala Zreiqat:** Writing – review & editing, Project administration, Conceptualization. **Vi Khanh Truong:** Writing – review & editing, Writing – original draft, Conceptualization.

## Declaration of competing interest

The authors declare that they have no known competing financial interests or personal relationships that could have appeared to influence the work reported in this paper.

## Data Availability

Data will be made available on request.

## References

[1] Koons G.L., Diba M., Mikos A.G. (2020). Materials design for bone-tissue engineering.

[2] Wu Q., Wang X., Jiang F., Zhu Z., Wen J., Jiang X. (2020). Study of Sr–Ca–Si-based scaffolds for bone regeneration in osteoporotic models.

[3] Kumar P., Dehiya B.S., Sindhu A. (2018). Bioceramics for hard tissue engineering applications: a review.

[4] Wang N., Fuh J.Y.H., Dheen S.T., Senthil Kumar A. (2021). Functions and applications of metallic and metallic oxide nanoparticles in orthopedic implants and scaffolds.

[5] Thippeswamy P.B., Nedunchelian M., Rajasekaran R.B., Riley D., Khatkar H., Rajasekaran S. (2021).

[6] Xie K., Wang N., Guo Y., Zhao S., Tan J., Wang L. (2022).

[7] Swain S., Padhy R.N., Rautray T.R. (2020).

[8] Nabiyouni M., Brückner T., Zhou H., Gbureck U., Bhaduri S.B. (2018).

[9] Johnson C.T., García A.J. (2015).

[10] Chen Z.-Y., Gao S., Zhang Y.-W., Zhou R.-B., Zhou F. (2021). Antibacterial biomaterials in bone tissue engineering.

[11] Beuriat P.A., Lohkamp L.N., Szathmari A., Rousselle C., Sabatier I., Di Rocco F., Mottolese C. (2019). Repair of Cranial bone defects in children using synthetic hydroxyapatite cranioplasty. CustomBone).

[12] G. N. Room. Medical implants market size to worth around US$ 145.6. https://www.globenewswire.com/news-release/2022/01/31/2375767/0/en/Medical-Implants-Market-Size-to-Worth-Around-US-145-6-Billion-by-2030.html.31 January 2022.

[13] Tangcharoensathien V., Sattayawutthipong W., Kanjanapimai S., Kanpravidth W., Brown R., Sommanustweechai A. (2017). Antimicrobial resistance: from global agenda to national strategic plan, Thailand. Bull. World Health Organ..

[14] Sumpradit N., Wongkongkathep S., Malathum K., Janejai N., Paveenkittiporn W., Yingyong T. (2021). Thailand's national strategic plan on antimicrobial resistance: progress and challenges. Bull. World Health Organ..

[15] Dadgostar P. (2019).

[16] Mahira S., Jain A., Khan W., Domb A.J. (2019).

[17] Lum Z.C., Natsuhara K.M., Shelton T.J., Giordani M., Pereira G.C., Meehan J.P. (2018). Mortality during total knee periprosthetic joint infection.

[18] Izakovicova P., Borens O., Trampuz A. (2019). Periprosthetic joint infection: current concepts and outlook.

[19] Sader H.S., Castanheira M., Streit J.M., Carvalhaes C.G., Mendes R.E. (2020). Frequency and antimicrobial susceptibility of bacteria causing bloodstream infections in pediatric patients from United States (US) medical centers (2014–2018): therapeutic options for multidrug-resistant bacteria.

[20] Vertes A., Hitchins V., Phillips K.S. (2012).

[21] Qin S., Xiao W., Zhou C., Pu Q., Deng X., Lan L. (2022). Pseudomonas aeruginosa: pathogenesis, virulence factors, antibiotic resistance, interaction with host, technology advances and emerging therapeutics.

[22] Ferraris S., Yamaguchi S., Barbani N., Cazzola M., Cristallini C., Miola M. (2020).

[23] Hench L.L., Jones J.R. (2015).

[24] Roohani-Esfahani S.-I., No Y.J., Lu Z., Ng P.Y., Chen Y., Shi J. (2016). A bioceramic with enhanced osteogenic properties to regulate the function of osteoblastic and osteocalastic cells for bone tissue regeneration.

[25] Zreiqat H., Ramaswamy Y., Wu C., Paschalidis A., Lu Z., James B. (2010). The incorporation of strontium and zinc into a calcium–silicon ceramic for bone tissue engineering.

[26] No Y.J., Nguyen T., Lu Z., Mirkhalaf M., Fei F., Foley M., Zreiqat H. (2021).

[27] Chen Y.-C., Hsu P.-Y., Tuan W.-H., Chen C.-Y., Wu C.-J., Lai P.-L. (2021).

[28] Tajvar S., Hadjizadeh A., Samandari S.S. (2023).

[29] Radunović A., Radunović O., Vulović M., Aksić M. (2023).

[30] Ma H., Feng C., Chang J., Wu C. (2018).

[31] Darus F., Isa R.M., Mamat N., Jaafar M. (2018). Techniques for fabrication and construction of three-dimensional bioceramic scaffolds: effect on pores size, porosity and compressive strength.

[32] Mirkhalaf M., Goldsmith J., Ren J., Dao A., Newman P., Schindeler A. (2021).

[33] Xu S., Wu Q., Guo Y., Ning C., Dai K. (2021).

[34] Pouroutzidou G.K., Papadopoulou L., Lazaridou M., Tsachouridis K., Papoulia C., Patsiaoura D. (2023). Composite PLGA–nanobioceramic coating on moxifloxacin-loaded akermanite 3D porous scaffolds for bone tissue regeneration.

[35] Qin H., Wei Y., Han J., Jiang X., Yang X., Wu Y. (2022). 3D printed bioceramic scaffolds: adjusting pore dimension is beneficial for mandibular bone defects repair.

[36] Fang Z., Chen J., Pan J., Liu G., Zhao C. (2021).

[37] Singh P., Yu X., Kumar A., Dubey A.K. (2022). Recent advances in silicate-based crystalline bioceramics for orthopedic applications: a review.

[38] Biegański P., Szczupak Ł., Arruebo M., Kowalski K. (2021). Brief survey on organometalated antibacterial drugs and metal-based materials with antibacterial activity.

[39] Fadeeva I.V., Lazoryak B.I., Davidova G.A., Murzakhanov F.F., Gabbasov B.F., Petrakova N.V. (2021). Antibacterial and cell-friendly copper-substituted tricalcium phosphate ceramics for biomedical implant applications.

[40] Gomes S., Vichery C., Descamps S., Martinez H., Kaur A., Jacobs A. (2018).

[41] Ofudje E.A., Adeogun A.I., Idowu M.A., Kareem S.O. (2019). Synthesis and characterization of Zn-Doped hydroxyapatite: scaffold application. antibacterial and bioactivity studies.

[42] He T., Chen H., Liu P., Shi H., Xu X., Feng C. (2023).

[43] Moghanian A., Sedghi A., Ghorbanoghli A., Salari E. (2018). The effect of magnesium content on in vitro bioactivity, biological behavior and antibacterial activity of sol–gel derived 58S bioactive glass.

[44] Predoi D., Iconaru S.L., Predoi M.V., Stan G.E., Buton N. (2019). Synthesis, characterization, and antimicrobial activity of magnesium-doped hydroxyapatite suspensions.

[45] Pham D.Q., Gangadoo S., Berndt C.C., Chapman J., Zhai J., Vasilev K. (2022). Antibacterial longevity of a novel Gallium liquid metal/hydroxyapatite composite coating fabricated by plasma spray.

[46] Mosina M., Siverino C., Stipniece L., Sceglovs A., Vasiljevs R., Moriarty T.F., Locs J. (2023). Gallium-doped hydroxyapatite shows antibacterial activity against Pseudomonas aeruginosa without affecting cell metabolic activity.

[47] Ibrahim A.M., Al-Rashidy Z.M., Abdel Ghany N.A., Ahmed H.Y., Omar A.E., Farag M.M. (2021). Bioactive and antibacterial metal implant composite coating based on Ce-doped nanobioactive glass and chitosan by electrophoretic deposition method.

[48] Guangjian D., Aili Y., Xiang C., Qingshan S., Ouyang Y., Shaozao T. (2012). Synthesis, characterization and antimicrobial activity of zinc and cerium co-doped α-zirconium phosphate.

[49] Bhattacharjee A., Gupta A., Verma M., Anand M.P., Sengupta P., Saravanan M. (2020). Antibacterial and magnetic response of site-specific cobalt incorporated hydroxyapatite.

[50] Chen F., Liu C., Mao Y. (2010). Bismuth-doped injectable calcium phosphate cement with improved radiopacity and potent antimicrobial activity for root canal filling.

[51] Ciobanu G., Harja M. (2019). Bismuth-doped nanohydroxyapatite coatings on Titanium implants for improved radiopacity and antimicrobial activity.

[52] Baheiraei N., Eyni H., Bakhshi B., Najafloo R., Rabiee N. (2021). Effects of strontium ions with potential antibacterial activity on in vivo bone regeneration.

[53] Anastasiou A., Nerantzaki M., Gounari E., Duggal M., Giannoudis P., Jha A., Bikiaris D. (2019). Antibacterial properties and regenerative potential of Sr2+ and Ce3+ doped fluorapatites; a potential solution for peri-implantitis.

[54] Tseng C.-F., Fei Y.-C., Chou Y.-J. (2020).

[55] Panneerselvam R., Anandhan N., Gopu G., Ganesan K., Marimuthu T. (2020).

[56] Sadeghzade S., Liu J., Wang H., Li X., Cao J., Cao H., Yuan H. (2022).

[57] Liu Z., He X., Chen S., Yu H. (2023).

[58] Xie C., Ye J., Liang R., Yao X., Wu X., Koh Y. (2021). Advanced strategies of biomimetic tissue‐engineered grafts for bone regeneration.

[59] Monfared M.H., Nemati A., Loghman F., Ghasemian M., Farzin A., Beheshtizadeh N., Azami M. (2022). A deep insight into the preparation of ceramic bone scaffolds utilizing robocasting technique.

[60] Tarín-Pelló A., Suay-García B., Pérez-Gracia M.-T. (2022). Antibiotic resistant bacteria: current situation and treatment options to accelerate the development of a new antimicrobial arsenal.

[61] Chen X., Zhou J., Qian Y., Zhao L. (2023).

[62] Mahanty A., Shikha D. (2022). Changes in the morphology, mechanical strength and biocompatibility of polymer and metal/polymer fabricated hydroxyapatite for orthopaedic implants. a review.

[63] Demir-Oğuz Ö., Boccaccini A.R., Loca D. (2023).

[64] Marin E., Boschetto F., Zanocco M., Honma T., Zhu W., Pezzotti G. (2021).

[65] Zhao C., Liu W., Zhu M., Wu C., Zhu Y. (2022). Bioceramic-based scaffolds with antibacterial function for bone tissue engineering. A review.

[66] Farazin A., Zhang C., Gheisizadeh A., Shahbazi A. (2023).

[67] Zhao C., Liu W., Zhu M., Wu C., Zhu Y. (2022).

[68] Liu Z., Yamada S., Otsuka Y., Galindo T.G.P., Tagaya M. (2022). Surface modification of hydroxyapatite nanoparticles for bone regeneration by controlling their surface hydration and protein adsorption states.

[69] Song W., Sun W., Chen L., Yuan Z. (2020).

[70] Sampath Kumar T., Madhumathi K. (2016). Handbook of Bioceramics and Biocomposites.

[71] Busscher H.J., van der Mei H.C., Subbiahdoss G., Jutte P.C., van den Dungen J.J., Zaat S.A. (2012). Biomaterial-associated infection: locating the finish line in the race for the surface.

[72] Ciofu O., Moser C., Jensen P.Ø., Høiby N. (2022). Tolerance and resistance of microbial biofilms.

[73] Fathima A., Arafath Y., Hassan S., Prathiviraj R., Kiran G.S., Selvin J. (2023). Understanding Microbial Biofilms.

[74] Kalelkar P.P., Riddick M., Garcia A.J. (2021).

[75] Masters E.A., Ricciardi B.F., Bentley K.L.d.M., Moriarty T.F., Schwarz E.M., Muthukrishnan G. (2022). Skeletal infections: microbial pathogenesis, immunity and clinical management.

[76] Musini A., Chilumoju S.P., Giri A. (2022). Microbial Biofilms.

[77] Speziale P., Pietrocola G., Foster T.J., Geoghegan J.A. (2014).

[78] Keane F.M., Loughman A., Valtulina V., Brennan M., Speziale P., Foster T.J. (2007). Fibrinogen and elastin bind to the same region within the A domain of fibronectin binding protein A, an MSCRAMM of Staphylococcus aureus.

[79] Peacock S.J., Foster T.J., Cameron B.J., Berendt A.R. (1999). Bacterial fibronectin-binding proteins and endothelial cell surface fibronectin mediate adherence of Staphylococcus aureus to resting human endothelial cells.

[80] Tristan A., Ying L., Bes M., Etienne J., Vandenesch F., Lina G. (2003). Use of multiplex PCR to identify Staphylococcus aureus adhesins involved in human hematogenous infections.

[81] Paharik A.E., Horswill A.R. (2016).

[82] McCourt J., O'Halloran D.P., McCarthy H., O'Gara J.P., Geoghegan J.A. (2014). Fibronectin-binding proteins are required for biofilm formation by community-associated methicillin-resistant Staphylococcus aureus strain LAC.

[83] A. Nouri, A. R. Shirvan, Y. Li, C. Wen. Smart Materials in Manufacturing.

[84] Adam B., Baillie G.S., Douglas L.J. (2002). Mixed species biofilms of Candida albicans and Staphylococcus epidermidis.

[85] Laverty G., Gorman S.P., Gilmore B.F. (2015).

[86] Blanco-Cabra N., Movellan J., Marradi M., Gracia R., Salvador C., Dupin D. (2022). Neutralization of ionic interactions by dextran-based single-chain nanoparticles improves tobramycin diffusion into a mature biofilm.

[87] Francolini I., Vuotto C., Piozzi A., Donelli G. (2017). Antifouling and antimicrobial biomaterials: an overview.

[88] Mas-Moruno C., Su B., Dalby M.J. (2019). Multifunctional coatings and nanotopographies: toward cell instructive and antibacterial implants.

[89] Ivanovski S., Bartold P.M., Huang Y.S. (2022). The role of foreign body response in peri‐implantitis: what is the evidence?.

[90] Kyriakides T.R., Kim H.-J., Zheng C., Harkins L., Tao W., Deschenes E. (2022). Foreign body response to synthetic polymer biomaterials and the role of adaptive immunity.

[91] Franz S., Rammelt S., Scharnweber D., Simon J.C. (2011). Immune responses to implants–a review of the implications for the design of immunomodulatory biomaterials.

[92] Hickok Noreen J., Li Bingyun, Oral Ebru, Zaat Sebastian A.J., Armbruster David A., Atkins Gerald J., Chen Antonia F. (2024). The 2023 Orthopedic Research Society’s international consensus meeting on musculoskeletal infection: Summary from the in vitro section. J. Orthopaed. Res..

[93] Croes M., van der Wal B.C.H., Vogely H.C. (2019). Impact of bacterial infections on osteogenesis: evidence from in vivo studies.

[94] Scherr T.D., Heim C.E., Morrison J.M., Kielian T. (2014). Hiding in plain sight: interplay between staphylococcal biofilms and host. Immunity.

[95] Robertson C.M., Perrone E.E., McConnell K.W., Dunne W.M., Boody B., Brahmbhatt T. (2008). Neutrophil depletion causes a fatal defect in murine pulmonary Staphylococcus aureus clearance.

[96] Brinkmann V., Reichard U., Goosmann C., Fauler B., Uhlemann Y., Weiss D.S. (2004). Neutrophil extracellular traps kill bacteria.

[97] Alder K.D., Lee I., Munger A.M., Kwon H.-K., Morris M.T., Cahill S.V. (2020). Intracellular Staphylococcus aureus in bone and joint infections: a mechanism of disease recurrence. inflammation, and bone and cartilage destruction.

[98] Amin Yavari S., Castenmiller S.M., van Strijp J.A., Croes M. (2020). Combating implant infections: shifting focus from bacteria to host.

[99] Kolaczkowska E., Kubes P. (2013). Neutrophil recruitment and function in health and inflammation.

[100] Vitkov L., Krautgartner W.D., Obermayer A., Stoiber W., Hannig M., Klappacher M., Hartl D. (2015). The initial inflammatory response to bioactive implants is characterized by NETosis.

[101] Kaplan S.S., Basford R.E., Jeong M.H., Simmons R.L. (1996). Biomaterial-neutrophil interactions: dysregulation of oxidative functions of fresh neutrophils induced by prior neutrophil-biomaterial interaction.

[102] van Kessel K.P., Bestebroer J., van Strijp J.A. (2014).

[103] Kou P.M., Babensee J.E. (2011). Macrophage and dendritic cell phenotypic diversity in the context of biomaterials.

[104] Vallés G., Bensiamar F., Maestro-Paramio L., García-Rey E., Vilaboa N., Saldaña L. (2020). Influence of inflammatory conditions provided by macrophages on osteogenic ability of mesenchymal. stem cells.

[105] Hamilton J.A. (2008). Colony-stimulating factors in inflammation and autoimmunity.

[106] Spiller K.L., Anfang R.R., Spiller K.J., Ng J., Nakazawa K.R., Daulton J.W., Vunjak-Novakovic G. (2014). The role of macrophage phenotype in vascularization of tissue engineering scaffolds.

[107] Gordon S., Taylor P.R. (2005). Monocyte and macrophage heterogeneity.

[108] Sica A., Mantovani A. (2012). Macrophage plasticity and polarization: in vivo veritas.

[109] Benoit M., Desnues B., Mege J.-L. (2008). Macrophage polarization in bacterial infections.

[110] Lawrence T., Natoli G. (2011). Transcriptional regulation of macrophage polarization: enabling diversity with identity.

[111] Chen Z., Klein T., Murray R.Z., Crawford R., Chang J., Wu C., Xiao Y. (2016). Osteoimmunomodulation for the development of advanced. bone biomaterials.

[112] Guan Y.-H., Wang N., Deng Z.-W., Chen X.-G., Liu Y. (2022).

[113] Wang S., Chen Y., Ling Z., Li J., Hu J., He F., Chen Q. (2022). The role of dendritic cells in the immunomodulation to implanted biomaterials.

[114] Cui Y., Liu H., Tian Y., Fan Y., Li S., Wang G. (2022).

[115] Verhoeven B.M., Mei S., Olsen T.K., Gustafsson K., Valind A., Lindström A. (2022). The immune cell atlas of human neuroblastoma.

[116] Cui Y., Li H., Li Y., Mao L. (2022). Novel insights into nanomaterials for immunomodulatory bone regeneration.

[117] Chung L., Maestas D.R., Housseau F., Elisseeff J.H. (2017).

[118] Yu F., Lian R., Liu L., Liu T., Bi C., Hong K. (2022). Biomimetic hydroxyapatite nanorods promote bone regeneration via accelerating osteogenesis of BMSCs through T cell-derived IL-22.

[119] Yu W.-w., Wan Q.-q., Wei Y., Li Y.-t., Li Q.-h., Ye T. (2022).

[120] Huang Y., Wu C., Zhang X., Chang J., Dai K. (2018).

[121] Moriarty T.F., Muthukrishnan G., Daiss J., Xie C., Nishitani K., Morita Y. (2021).

[122] Barrak F.N., Li S., Mohammed A.A., Myant C., Jones J.R. (2022).

[123] Sadowska J.M., Ginebra M.-P. (2020). Inflammation and biomaterials: role of the immune response in bone regeneration by inorganic scaffolds.

[124] Clézardin P., Coleman R., Puppo M., Ottewell P., Bonnelye E., Paycha F. (2021). Bone metastasis: mechanisms, therapies, and biomarkers.

[125] Soriente A., Fasolino I., Gomez‐Sánchez A., Prokhorov E., Buonocore G.G., Luna‐Barcenas G. (2022). Chitosan/hydroxyapatite nanocomposite scaffolds to modulate osteogenic and inflammatory response.

[126] Xiao L., Shiwaku Y., Hamai R., Tsuchiya K., Sasaki K., Suzuki O. (2021). Macrophage polarization related to crystal phases of calcium phosphate biomaterials.

[127] Velard F., Braux J., Amedee J., Laquerriere P. (2013). Inflammatory cell response to calcium phosphate biomaterial particles: an overview.

[128] Sadowska J.M., Wei F., Guo J., Guillem-Marti J., Lin Z., Ginebra M.-P., Xiao Y. (2019).

[129] Liu X., Ouyang L., Chen L., Qiao Y., Ma X., Xu G., Liu X. (2022).

[130] Mahon O.R., O'Hanlon S., Cunningham C.C., McCarthy G.M., Hobbs C., Nicolosi V. (2018).

[131] Raja F.N.S., Worthington T., Isaacs M.A., Rana K.S., Martin R.A. (2019).

[132] Chitra S., Bargavi P., Balasubramaniam M., Chandran R.R., Balakumar S. (2020).

[133] Jaiswal S., McHale P., Duffy B. (2012).

[134] Guldiren D., Aydın S. (2017).

[135] Samani S., Hossainalipour S., Tamizifar M., Rezaie H. (2013). In Vitro antibacterial evaluation of sol–gel‐derived Zn‐, Ag‐, and (Zn+ Ag)‐doped hydroxyapatite coatings against methicillin‐resistant Staphylococcus aureus.

[136] Schatkoski V.M., do Amaral Montanheiro T.L., de Menezes B.R.C., Pereira R.M., Rodrigues K.F., Ribas R.G. (2021). Current advances concerning the most cited metal ions doped bioceramics and silicate-based bioactive glasses for bone tissue engineering. Ceram. Int..

[137] Linder M.C., Hazegh-Azam M. (1996). Copper biochemistry and molecular biology.

[138] Rigo A., Corazza A., di Paolo M.L., Rossetto M., Ugolini R., Scarpa M. (2004). Interaction of copper with cysteine: stability of cuprous complexes and catalytic role of cupric ions in anaerobic thiol oxidation.

[139] Festa R.A., Thiele D.J. (2011). Copper: an essential metal in biology.

[140] Scarpa M., Momo F., Viglino P., Vianello F., Rigo A. (1996). Activated oxygen species in the oxidation of glutathione A kinetic study.

[141] Silver S., Phung L.T. (2005). A bacterial view of the periodic table: genes and proteins for toxic inorganic ions.

[142] Franke S., Grass G., Rensing C., Nies D.H. (2003). Molecular analysis of the copper-transporting efflux system CusCFBA of Escherichia coli.

[143] Abicht H.K., Gonskikh Y., Gerber S.D., Solioz M. (2013). Non-enzymic copper reduction by menaquinone enhances copper toxicity in Lactococcus lactis IL1403.

[144] Calvano C., Picca R., Bonerba E., Tantillo G., Cioffi N., Palmisano F. (2016). MALDI‐TOF mass spectrometry analysis of proteins and lipids in Escherichia coli exposed to copper ions and nanoparticles.

[145] Liu J., Hurt R.H. (2010). Ion release kinetics and particle persistence in aqueous nano-silver colloids.

[146] Zhang S., Du C., Wang Z., Han X., Zhang K., Liu L. (2013). Reduced cytotoxicity of silver ions to mammalian cells at high concentration due to the formation of silver chloride.

[147] Rai M., Deshmukh S.D., Ingle A.P., Gupta I.R., Galdiero M., Galdiero S. (2016). Metal nanoparticles: the protective nanoshield against virus infection.

[148] Kędziora A., Speruda M., Krzyżewska E., Rybka J., Łukowiak A., Bugla-Płoskońska G. (2018). Similarities and differences between silver ions and silver in nanoforms as antibacterial agents.

[149] Nguyen Tien Thanh, Zhang Pengfei, Bi Jingwei, Nguyen Ngoc Huu, Dang Yen, Xu Zhaoning, Wang Hao (2023). Silver─ Gallium nano‐amalgamated particles as a novel, biocompatible solution for antibacterial coatings. Adv. Function. Mater.

[150] Bondarenko O.M., Sihtmäe M., Kuzmičiova J., Ragelienė L., Kahru A., Daugelavičius R. (2018).

[151] Shi C., Gao J., Wang M., Shao Y., Wang L., Wang D., Zhu Y. (2016). Functional hydroxyapatite bioceramics with excellent osteoconductivity and stern-interface induced antibacterial ability.

[152] Pham D., Gangadoo S., Lu Z., Berndt C., Newsom E., Zreiqat H. (2022).

[153] Ikram M., Shahzadi A., Bilal M., Haider A., Ul-Hamid A., Nabgan W. (2023). Strontium-doped chromium oxide for RhB reduction and antibacterial activity with evidence of molecular docking analysis.

[154] Marcello E., Maqbool M., Nigmatullin R., Cresswell M., Jackson P.R., Basnett P. (2021).

[155] Jia F., Xu D., Sun Y., Jiang W., Yang H., Bian A. (2023). Strontium-calcium doped titanium dioxide nanotubes loaded with GL13K for promotion of antibacterial activity. anti-Inflammation, and vascularized bone regeneration.

[156] O'Sullivan C., O'Neill L., O'Leary N.D., O'Gara J.P., Crean A.M., Osteointegration K. B. Ryan (2021).

[157] Degli Esposti L., Ionescu A.C., Carella F., Adamiano A., Brambilla E., Iafisco M. (2022).

[158] Makvandi P., Wang C.y., Zare E.N., Borzacchiello A., Niu L.n., Tay F.R. (2020). Metal‐based nanomaterials in biomedical applications: antimicrobial activity and cytotoxicity aspects.

[159] McDevitt C.A., Ogunniyi A.D., Valkov E., Lawrence M.C., Kobe B., McEwan A.G., Paton J.C. (2011). A molecular mechanism for bacterial susceptibility to zinc.

[160] Siddiqi K.S., ur Rahman A., Husen A. (2018). Properties of zinc oxide nanoparticles and their activity against microbes.

[161] Xia P., Lian S., Wu Y., Yan L., Quan G., Zhu G. (2021). Zinc is an important inter-kingdom signal between the host and microbe.

[162] Belenky P., Jonathan D.Y., Porter C.B., Cohen N.R., Lobritz M.A., Ferrante T. (2015). Bactericidal antibiotics induce toxic metabolic perturbations that lead to cellular damage.

[163] Godoy-Gallardo M., Eckhard U., Delgado L.M., de Roo Puente Y.J., Hoyos-Nogués M., Gil F.J., Perez R.A. (2021). Antibacterial approaches in tissue engineering using metal ions and nanoparticles: from mechanisms to applications.

[164] Kurtjak M., Vukomanović M., Krajnc A., Kramer L., Turk B., Suvorov D. (2016). Designing Ga (iii)-containing hydroxyapatite with antibacterial activity.

[165] Mathews S., Hans M., Mücklich F., Solioz M. (2013). Contact killing of bacteria on copper is suppressed if bacterial-metal contact is prevented and is induced on iron by copper ions.

[166] Lemire J.A., Harrison J.J., Turner R.J. (2013). Antimicrobial activity of metals: mechanisms, molecular targets and applications.

[167] Stohs S.J., Bagchi D. (1995). Oxidative mechanisms in the toxicity of metal ions.

[168] Macomber L., Rensing C., Imlay J.A. (2007). Intracellular copper does not catalyze the formation of oxidative DNA damage in Escherichia coli.

[169] Anjem A., Varghese S., Imlay J.A. (2009). Manganese import is a key element of the OxyR response to hydrogen peroxide in Escherichia coli.

[170] Xu F.F., Imlay J.A. (2012). Silver (I), mercury (II), cadmium (II), and zinc (II) target exposed enzymic iron-sulfur clusters when they toxify Escherichia coli.

[171] Xu H., Qu F., Xu H., Lai W., Andrew Wang Y., Aguilar Z.P., Wei H. (2012). Role of reactive oxygen species in the antibacterial mechanism of silver nanoparticles on Escherichia coli O157: H7.

[172] Valko M., Morris H., Cronin M. (2005). Metals, toxicity and oxidative stress.

[173] Bhakta J.N. (2017). Metal toxicity in microorganism. Handbook of research on inventive bioremediation techniques. IGI Global.

[174] Lemire J.A., Turner R.J. (2016).

[175] Imlay J.A. (2014). The mismetallation of enzymes during oxidative stress.

[176] Chitambar C.R. (2016). Gallium and its competing roles with iron in biological systems.

[177] Sumner E.R., Shanmuganathan A., Sideri T.C., Willetts S.A., Houghton J.E., Avery S.V. (2005). Oxidative protein damage causes chromium toxicity in yeast.

[178] Amici A., Levine R., Tsai L., Stadtman E. (1989). Conversion of amino acid residues in proteins and amino acid homopolymers to carbonyl derivatives by metal-catalyzed oxidation reactions.

[179] Palza H. (2015). Antimicrobial polymers with metal nanoparticles.

[180] Warnes S.L., Highmore C.J., Keevil C.W. (2012). Horizontal transfer of antibiotic resistance genes on abiotic touch surfaces: implications for public health.

[181] Linley E., Denyer S.P., McDonnell G., Simons C., Maillard J.-Y. (2012). Use of hydrogen peroxide as a biocide: new consideration of its mechanisms of biocidal action.

[182] Warnes S., Keevil C. (2011). Mechanism of copper surface toxicity in vancomycin-resistant enterococci following wet or dry surface contact.

[183] Warnes S.L., Keevil C.W. (2016). Lack of involvement of Fenton chemistry in death of methicillin-resistant and methicillin-sensitive strains of Staphylococcus aureus and destruction of their genomes on wet or dry copper alloy surfaces.

[184] Wang M., Tang T. (2019).

[185] Campoccia D., Montanaro L., Arciola C.R. (2013). A review of the biomaterials technologies for infection-resistant surfaces.

[186] Raphel J., Holodniy M., Goodman S.B., Heilshorn S.C. (2016).

[187] Hazell G., Fisher L.E., Murray W.A., Nobbs A.H., Su B. (2018).

[188] Tuson H.H., Weibel D.B. (2013). Bacteria–surface interactions.

[189] Crawford R.J., Webb H.K., Truong V.K., Hasan J., Ivanova E.P. (2012). Surface topographical factors influencing bacterial attachment.

[190] Oh J.K., Yegin Y., Yang F., Zhang M., Li J., Huang S. (2018). The influence of surface chemistry on the kinetics and thermodynamics of bacterial adhesion.

[191] Yuan Y., Hays M.P., Hardwidge P.R., Kim J. (2017). Surface characteristics influencing bacterial adhesion to polymeric substrates.

[192] Yoda I., Koseki H., Tomita M., Shida T., Horiuchi H., Sakoda H., Osaki M. (2014). Effect of surface roughness of biomaterials on Staphylococcus epidermidis adhesion.

[193] Ammar Y., Swailes D., Bridgens B., Chen J. (2015).

[194] Wassmann T., Kreis S., Behr M., Buergers R. (2017). The influence of surface texture and wettability on initial bacterial adhesion on titanium and zirconium oxide dental implants.

[195] Dutra D., Pereira G., Kantorski K., Exterkate R., Kleverlaan C., Valandro L., Zanatta F. (2017). Grinding with diamond burs and hydrothermal aging of a Y-TZP material: effect on the material surface characteristics and bacterial adhesion.

[196] Kang D.-H., Choi H., Yoo Y.-J., Kim J.-H., Park Y.-B., Moon H.-S. (2017). Effect of polishing method on surface roughness and bacterial adhesion of zirconia-porcelain veneer.

[197] Sarker A., Tran N., Rifai A., Brandt M., Tran P.A., Leary M. (2019).

[198] Lu A., Gao Y., Jin T., Luo X., Zeng Q., Shang Z. (2020). Effects of surface roughness and texture on the bacterial adhesion on the bearing surface of bio-ceramic joint implants: an in vitro study.

[199] Edwards K.J., Rutenberg A.D. (2001). Microbial response to surface microtopography: the role of metabolism in localized mineral dissolution.

[200] Liu J., Ford R.M. (2009). Idling time of swimming bacteria near particulate surfaces contributes to apparent adsorption coefficients at the macroscopic scale under static conditions.

[201] Rieger K., Thyagarajan R., Hoen M., Yeung H., Ford D., Schiffman J. (2016). Transport of microorganisms into cellulose nanofiber mats.

[202] Aykent F., Yondem I., Ozyesil A.G., Gunal S.K., Avunduk M.C., Ozkan S. (2010). Effect of different finishing techniques for restorative materials on surface roughness and bacterial adhesion.

[203] Thio B.J.R., Meredith J.C. (2008). Quantification of E. coli adhesion to polyamides and polystyrene with atomic force microscopy.

[204] Thomas W.E., Trintchina E., Forero M., Vogel V., Sokurenko E.V. (2002). Bacterial adhesion to target cells enhanced by shear force.

[205] Damiati L., Eales M.G., Nobbs A.H., Su B., Tsimbouri P.M., Salmeron-Sanchez M., Dalby M.J. (2018).

[206] Tallawi M., Opitz M., Lieleg O. (2017). Modulation of the mechanical properties of bacterial biofilms in response to environmental challenges.

[207] Katsikogianni M., Spiliopoulou I., Dowling D., Missirlis Y. (2006). Adhesion of slime producing Staphylococcus epidermidis strains to PVC and diamond-like carbon/silver/fluorinated coatings.

[208] Rowland B.M. (2003). Bacterial contamination of dental unit waterlines: what is your dentist spraying into your mouth?.

[209] Kim H.-J., Tango C.N., Chelliah R., Oh D.-H. (2019). Sanitization efficacy of slightly acidic electrolyzed water against pure cultures of Escherichia coli, Salmonella enterica, Typhimurium, Staphylococcus aureus and Bacillus cereus spores. comparison with different water hardness.

[210] Ribeiro M., Monteiro F.J., Ferraz M.P. (2012). Infection of orthopedic implants with emphasis on bacterial adhesion process and techniques used in studying bacterial-material interactions.

[211] Kinnari T.J., Esteban J., Martin-de-Hijas N.Z., Sánchez-Muñoz O., Sánchez-Salcedo S., Colilla M. (2009). Influence of surface porosity and pH on bacterial adherence to hydroxyapatite and biphasic calcium phosphate bioceramics.

[212] Pelepenko L.E., Saavedra F., Antunes T.B.M., Bombarda G.F., Gomes B., Zaia A.A. (2021). Physicochemical, antimicrobial, and biological properties of White-MTAFlow.

[213] Huang Y., Li X., Mandal P., Wu Y., Liu L., Gui H., Liu J. (2019). The in vitro antimicrobial activities of four endodontic sealers.

[214] Khoo X., Grinstaff M.W. (2011). Novel infection-resistant surface coatings: a bioengineering approach.

[215] Boda S.K., Basu B. (2017). Engineered biomaterial and biophysical stimulation as combinatorial strategies to address prosthetic infection by pathogenic bacteria.

[216] Horváthy D.B., Simon M., Schwarz C.M., Masteling M., Vácz G., Hornyák I., Lacza Z. (2017). Serum albumin as a local therapeutic agent in cell therapy and tissue engineering.

[217] Wronska M.A., O'Connor I.B., Tilbury M.A., Srivastava A., Wall J.G. (2016). Adding functions to biomaterial surfaces through protein incorporation.

[218] Yang Z., Liu M., Yang Y., Zheng M., Liu X., Tan J. (2020). Biofunctionalization of zirconia with cell-adhesion peptides via polydopamine crosslinking for soft tissue engineering: effects on the biological behaviors of human gingival fibroblasts and oral bacteria.

[219] Eroshenko D., Morozov I., Korobov V. (2015). The role of plasma, albumin, and fibronectin in Staphylococcus epidermidis adhesion to polystyrene surface.

[220] Kamarudin N.H.A., Rahman R.N.Z.R.A., Ali M.S.M., Leow T.C., Basri M., Salleh A.B. (2014). Unscrambling the effect of C-terminal tail deletion on the stability of a cold-adapted, organic solvent stable lipase from Staphylococcus epidermidis AT2.

[221] Therrien A., Fournier A., Lafleur M. (2016). Role of the cationic C-terminal segment of melittin on membrane fragmentation.

[222] Vasconcelos D.M., Falentin-Daudré C., Blanquaert D., Thomas D., Granja P.L., Migonney V. (2014). Role of protein environment and bioactive polymer grafting in the S. epidermidis response to titanium alloy for biomedical applications.

[223] Arciola C.R., Campoccia D., Gamberini S., Donati M.E., Montanaro L. (2004). Presence of fibrinogen-binding adhesin gene in Staphylococcus epidermidis isolates from central venous catheters-associated and orthopaedic implant-associated infections.

[224] Charville G.W., Hetrick E.M., Geer C.B., Schoenfisch M.H. (2008). Reduced bacterial adhesion to fibrinogen-coated substrates via nitric oxide release.

[225] Pei L., Flock J.-I. (2001). Functional study of antibodies against a fibrogenin-binding protein in Staphylococcus epidermidis adherence to polyethylene catheters.

[226] Vallet‐Regí M., Lozano D., González B., Izquierdo‐Barba I. (2020). Biomaterials against bone infection.

[227] de Lima G.G., Campos L., Junqueira A., Devine D.M., Nugent M.J. (2015). A novel pH‐sensitive ceramic‐hydrogel for biomedical applications.

[228] Chakraborty S. (2023).

[229] Li P., Jia Z., Wang Q., Tang P., Wang M., Wang K. (2018). A resilient and flexible chitosan/silk cryogel incorporated Ag/Sr co-doped nanoscale hydroxyapatite for osteoinductivity and antibacterial properties.

[230] Faruq O., Sayed S., Kim B., Im S.B., Lee B.T. (2020). A biphasic calcium phosphate ceramic scaffold loaded with oxidized cellulose nanofiber–gelatin hydrogel with immobilized simvastatin drug for osteogenic differentiation.

[231] Colilla M., Vallet-Regí M. (2020). Targeted stimuli-responsive mesoporous silica nanoparticles for bacterial infection treatment.

[232] Sadtler K., Singh A., Wolf M.T., Wang X., Pardoll D.M., Design J. H. Elisseeff (2016). Clinical translation and immunological response of biomaterials in regenerative medicine.

[233] Tofail S.A., Koumoulos E.P., Bandyopadhyay A., Bose S., O'Donoghue L., Charitidis C. (2018). Additive manufacturing: scientific and technological challenges, market uptake and opportunities.

[234] Place E.S., Evans N.D., Stevens M.M. (2009). Complexity in biomaterials for tissue engineering.

[235] Ur Rehman M.A., Ferraris S., Goldmann W.H., Perero S., Bastan F.E., Nawaz Q. (2017). Antibacterial and bioactive coatings based on radio frequency co-sputtering of silver nanocluster-silica coatings on PEEK/bioactive glass layers obtained by electrophoretic deposition.

[236] Baino F., Verné E., Fiume E., Peitl O., Zanotto E.D., Brandão S.M., Schellini S.A. (2019). Bioactive glass and glass‐ceramic orbital implants.

